# Supplementary material for: Epidermal Growth Factor Receptor Expression Licenses Type-2 Helper T Cells to Function in a T Cell Receptor-Independent Fashion
Source: Immunity. 2017 Oct 17;47(4):710–722.e6. doi: 10.1016/j.immuni.2017.09.013 (PMC5654729; doi:10.1016/j.immuni.2017.09.013)
Supplement: Document S1. Figures S1–S7 [file mmc1.pdf]

Immunity, Volume *47*

Supplemental Information

Epidermal Growth Factor Receptor Expression Licenses Type-2 Helper T Cells to Function in a T Cell Receptor-Independent Fashion

Carlos M. Minutti, Sebastian Drube, Natalie Blair, Christian Schwartz, Jame C. McCrae, Andrew N. McKenzie, Thomas Kamradt, Michal Mokry, Paul J. Coffer, Maria Sibilia, Alice J. Sijts, Padraic G. Fallon, Rick M. Maizels, and Dietmar M. Zaiss

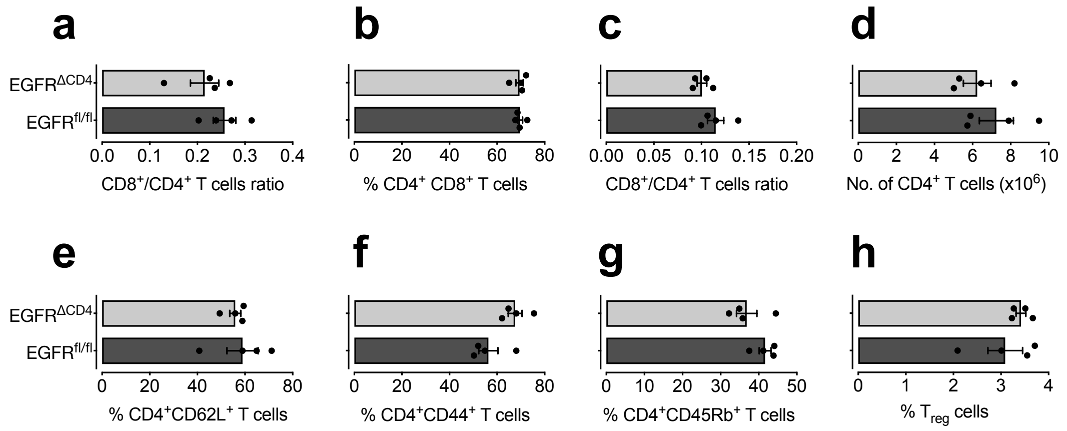

Supplementary Figure S1. Related to Figure 1.

Characterization of naive EGFR$^{\Delta CD4}$ mice. Thymi and spleens were harvested from naïve *wt* and EGFR$^{\Delta CD4}$ mice. **a)** Ratio of CD4 and CD8 single positive T-cells and **b)** frequency of CD4, CD8 double positive T-cells from thymus was determined by FACS. **c)** Ratio of CD4$^+$ and CD8$^+$ positive T-cells and **d)** frequency of CD4$^+$ T-cells from spleens as determined by FACS. Characterization of different CD4$^+$ T-cell sub-populations from spleens: **e)** CD62L$^+$, **f)** CD44$^+$, **g)** CD45Rb$^+$ and **h)** CD25$^+$ FoxP3$^+$ regulatory T-cells. Data are representative of two independent experiments (mean ± SEM); results for individual mice are shown as dots.

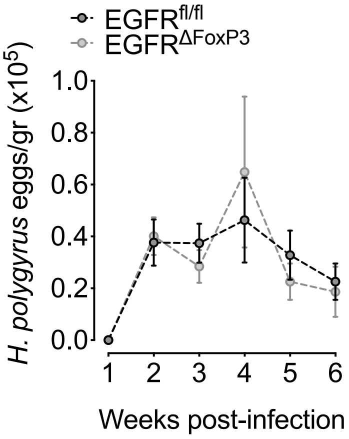

Supplementary Figure S2. Related to Figure 1.

EGFR expression by regulatory T cells does not contribute to worm clearance. WT and EGFR$^{\Delta FoxP3}$ mice were infected with *H. polygyrus* larvae or left untreated. Egg count in faeces were analysed at different times following infection with *H. polygyrus* (n = 3 mice). Data are representative of two independent experiments (mean ± SEM).

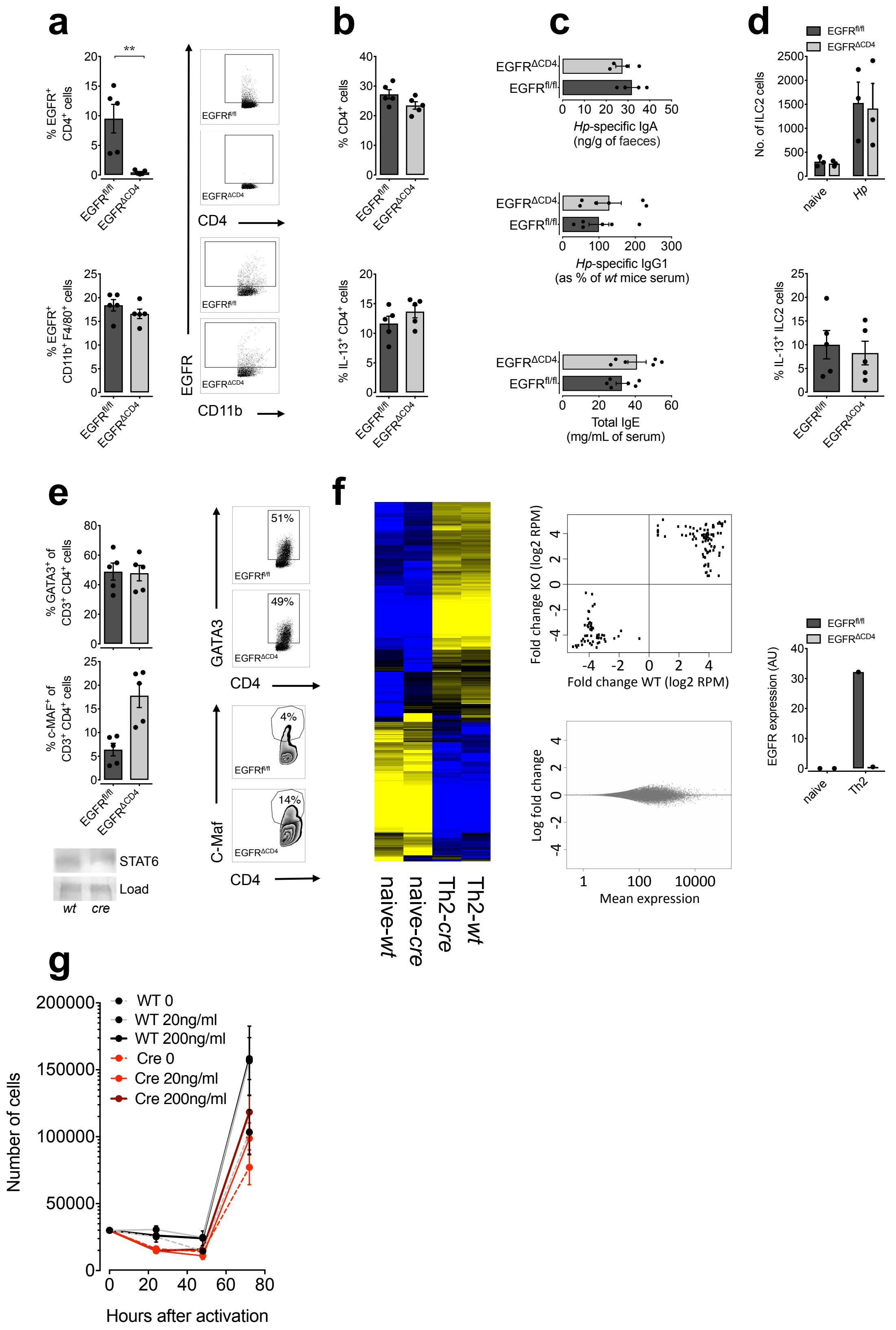
a
b
c
d
e
f
g
% EGFR+ CD4+ cells
% EGFR+ CD11b+ F4/80+ cells
EGFR
CD4
CD11b
% CD4+ cells
% IL-13+ CD4+ cells
Hp-specific IgA (ng/g of faeces)
Hp-specific IgG1 (as % of wt mice serum)
Total IgE (mg/mL of serum)
No. of ILC2 cells
% IL-13+ ILC2 cells
naive
Hp
% GATA3+ of CD3+ CD4+ cells
% c-MAF+ of CD3+ CD4+ cells
GATA3
C-Maf
51%
49%
4%
14%
STAT6
Load
wt
cre
naive-wt
naive-cre
Th2-cre
Th2-wt
Fold change KO (log2 RPM)
Fold change WT (log2 RPM)
Log fold change
Mean expression
EGFR expression (AU)
Th2
WT 0
WT 20ng/ml
WT 200ng/ml
Cre 0
Cre 20ng/ml
Cre 200ng/ml
Number of cells
Hours after activation

Supplementary Figure S3. Related to Figure 1.

Characterization of the immune response of EGFR$^{\Delta CD4}$ mice to *H. polygyrus* infection. a-d) *wt* and EGFR$^{\Delta CD4}$ mice were infected with *H. polygyrus* larvae or left untreated. Samples were collected on day 14 after infection. **a)** FACS analysis of the expression of EGFR on CD4^{+} T-cells **(upper)** and macrophages **(lower)** from mLNs. **b)** Frequency of total CD4^{+}T helper **(upper)** and Th2 **(lower)** cells from mLNs of infected mice. **c)** Total IgE and *H. polygyrus*-specific, IgA and IgG1 titers in faeces and sera of infected mice. **d)** Frequency of ILC2 from mLNs of naïve and infected **(upper)** mice and the capability of ILC2 from mLNs of infected mice to induce IL-13 in response to IL-33 **(lower)**. **e-f)** Th2 cells from *wt* and EGFR$^{\Delta CD4}$ mice were generated in vitro by adding IL-2, IL-4 and neutralizing IFN-γ antibody into the culture**. e)** The expression of GATA3 and c-Maf was determined by FACS analysis and the expression of STAT6 was determined by WB. **f)** RNA-seq analysis of *wt* and EGFR-deficient in vitro differentiated Th2 cells before and after 4 day differentiation. Heatmap shows median centered, log2 RPKM gene expression values with yellow depicting maximal z-score of 3 log2 RPKM and blue depicting z-score of -3 log2 RPKM. MA plot shows (lack of) genotype specific gene expression changes after 4 day differentiation. **g)** The proliferation in response to various concentrations of IL-2 was compared between naïve T cells from *wt* and EGFR$^{\Delta CD4}$ mice. $^{**}P < 0.01$, compared as indicated (Two-tailed Mann–Whitney test). Data are representative of two independent experiments (mean ± SEM); results for individual mice are shown as dots.

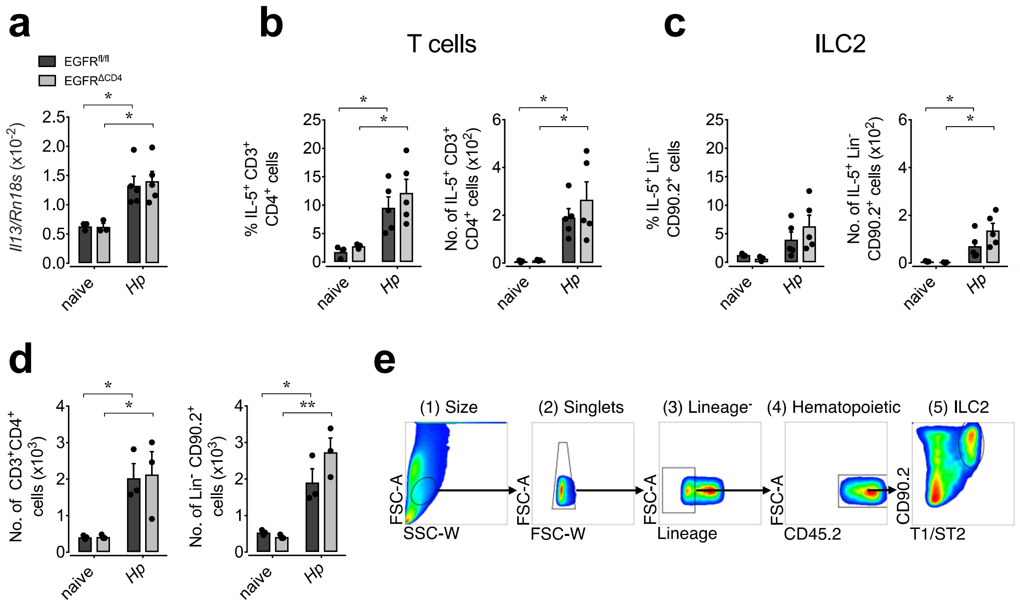

Supplementary Figure S4. Related to Figure 2.

Characterization of the cellular sources of IL-13 at the site of infection. *wt* and EGFR$^{\Delta CD4}$ mice were infected with *H. polygyrus* larvae or left untreated and samples were collected at day 8 post-infection. **a)** *Il13* was analyzed in the mLNs of naïve and infected mice by RT-PCR. **b-e)** Mice were treated with BFA 6 hours prior to harvest, after which, single cell preparations from duodena of naïve or infected mice were prepared and analyzed by FACS: Percentage and absolute number of IL-5 expressing T cells (**b**) and ILC-2 (**c**) as well as total T cells and ILC-2 **(d)**. **e)** Sequential identification of ILC-2 by flow cytometry. Cells are first identified by their size (1), and then as singlets (2). Lin− cells (3) are obtained by gating out CD3$^+$, CD11b$^+$, CD11c$^+$, FcεR1$^+$, CD19$^+$, and NK1.1$^+$ cells. Subsequently, hematopoietic cells are selected by their expression of CD45.2 (4). Finally, Lin− and CD45.2$^+$ populations are further subgated on the basis of the expression of CD90.2 versus T1/ST2. CD90.2$^+$ and T1/ST2$^+$ cells are what we defined as ILC-2 (5). All shown gates are children of the parent gates shown previously. In some experiments, CD3 was removed from the Lineage cocktail and used with a different conjugate in order to co-stain T-cells (CD3$^+$ CD4$^+$) and ILC-2. *$P < 0.05$, **$P < 0.01$ and ***$P < 0.001$ compared as indicated (ANOVA with the Tukey-Kramer HSD multiple-comparison test). Data are representative of two independent experiments (mean ± SEM); results for individual mice are shown as dots.

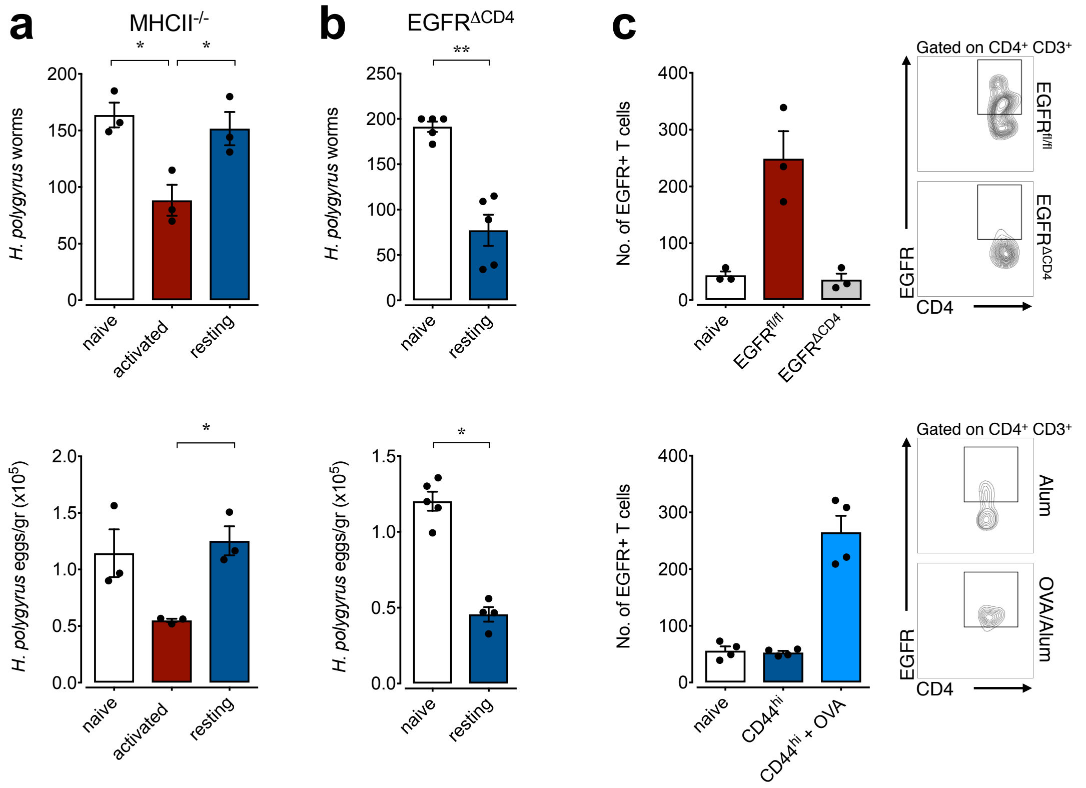

Supplementary Figure S5. Related to Figure 5.

Activated but not resting T-cells provide protection independently of TCR signaling. a) MHC-II$^{-/-}$ and **b)** EGFR$^{\Delta CD4}$ mice were infected with *H. polygyrus* larvae and 7 days post-infection received purified CD4^{+} T-cells derived from mLNs of naïve or *wt* mice that were infected with *H. polygyrus* for two weeks (activated in red) or from *H. polygyrus*-infected mice that had been treated with pyrantel embonate two weeks prior to transfer (resting in blue). Worm burden (**upper**) and egg counts in faeces (**lower**) were analysed 2 weeks post-transfer. **c)** EGFR$^{\Delta CD4}$ mice were infected with *H. polygyrus* larvae and received either FACS-sorted CD4^{+} CD69^{+} activated T-cells **(upper)** or FACS-sorted memory T-cells **(lower)** from mice that were challenged intra-nasally with OVA. EGFR expressing cells were recovered in single cells suspensions from the duodena of recipient mice and analyzed by FACS. *P < 0.05 and **P < 0.01, compared as indicated (Mann-Whitney U test). Data are representative of two independent experiments (except from c) (mean ± SEM); results for individual mice are shown as dots.

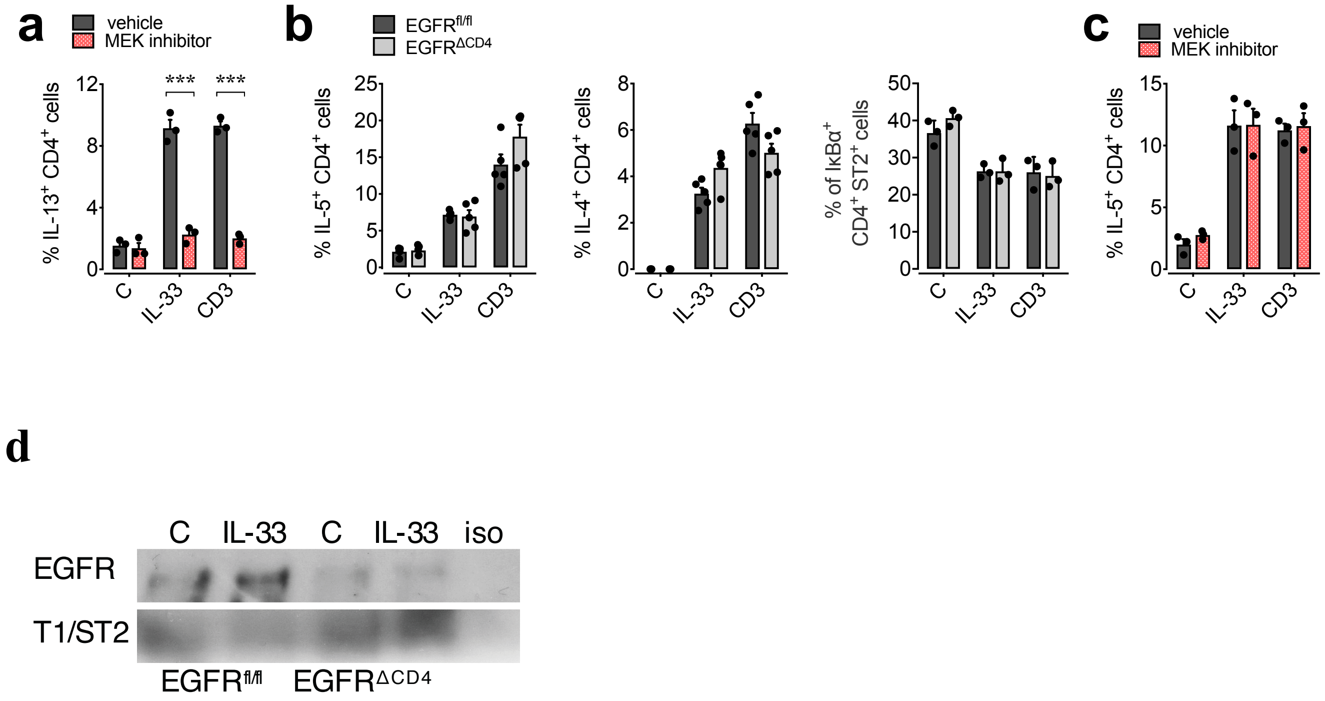

Supplementary Figure S6. Related to Figure 6.

Characterization of the signaling pathways required to induce IL-13 and IL-5 by Th2 cells. *wt* and EGFR$^{\Delta CD4}$ mice were infected with *H. polygyrus* larvae and on day 14 post-infection mLN were harvested. mLN cells were stimulated with IL-33, anti-CD3 or media in the presence **(a,c)** or absence **(b)** of MEK inhibitor (10 μM) (orange bars) and vehicle. **a)** Expression of IL-13 in *wt* T cells treated with MEK inhibitor was determined by FACS. **b**) Expression of IL-5, IL-4 and IκBα was determined by FACS analysis in *wt* and EGFR$^{\Delta CD4}$ T-cells. **c)** Expression of IL-5 in *wt* T cells treated with MEK inhibitor was determined by FACS. **d**) T1/ST2 was immune-precipitated from MACS-enriched *wt* and EGFR$^{\Delta CD4}$ CD4 T-cells and the presence of EGFR in the immuno-complexes was analyzed by Western blot.

***P < 0.001, compared as indicated (ANOVA with the Tukey-Kramer HSD multiple-comparison test). All data are representative of at least two independent experiments (mean ± SEM); results for individual mice are shown as dots.

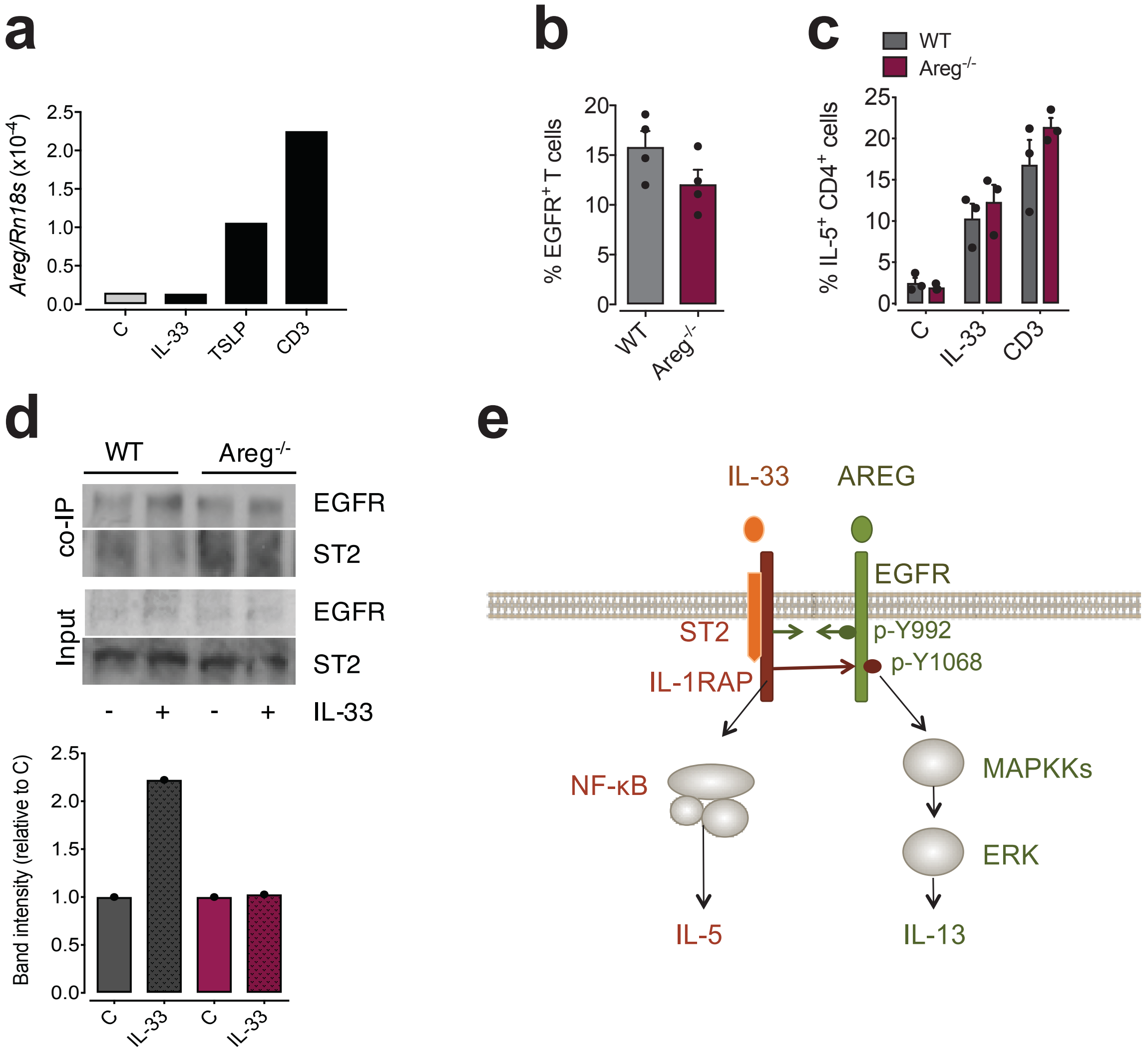

Supplementary Figure S7. Related to Figure 7.

Characterization of the function of AREG during the formation of an EGFR/ST2 signaling complex on the surface of Th2 cells. *wt* and *Areg*$^{-/-}$ mice were infected with *H. polygyrus* larvae and on day 14 post-infection mLN were harvested. **a)** AREG expression following different stimuli **b)** Comparison of EGFR expression on mLN CD4^{+} T cells from *H. polygyrus*-infected *wt* and *Areg*$^{-/-}$ mice. **c)** mLN cells were stimulated with IL-33, anti-CD3 or media only and expression of IL-5 was determined by FACS analysis. **d)** T1/ST2 was immune-precipitated from FACS sorted *wt* and *Areg*$^{-/-}$ CD4 T-cells and the presence of EGFR in the immuno-complexes was analyzed by Western blot (**upper**) and band intensity per lane determined (**lower**). **e)** Scheme explaining how AREG-induced Y-992 phosphorylation of the EGFR on Th2 cells allows for the formation of an active signaling complex between the EGFR and T1/ST2.

All data are representative of at least two independent experiments (mean ± SEM); results for individual mice are shown as dots.
