# Supplementary material for: Epidermal Growth Factor Receptor Expression Licenses Type-2 Helper T Cells to Function in a T Cell Receptor-Independent Fashion
Source: Immunity. 2017 Oct 17;47(4):710–722.e6. doi: 10.1016/j.immuni.2017.09.013 (PMC5654729; doi:10.1016/j.immuni.2017.09.013)
Supplement: Document S2. Article plus Supplemental Information [file mmc2.pdf]

Immunity

Article

Epidermal Growth Factor Receptor Expression Licenses Type-2 Helper T Cells to Function in a T Cell Receptor-Independent Fashion

Graphical Abstract

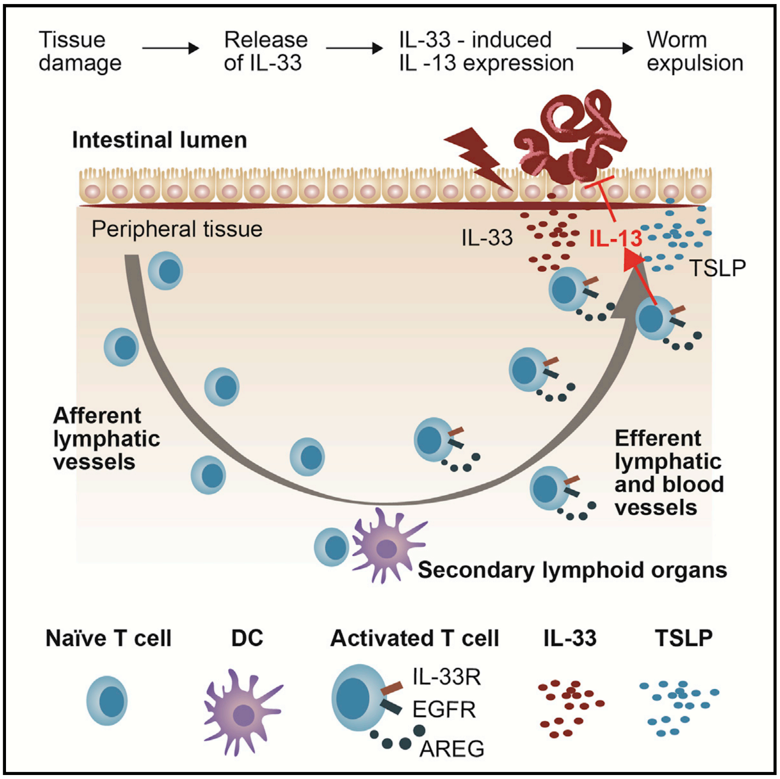

Highlights

- Mice lacking EGFR expression on T cells are more susceptible to worm infections
- EGFR forms a complex with T1/ST2, allowing for IL-33 induced IL-13 expression
- Amphiregulin-mediated EGFR activation is essential for complex formation with T1/ST2
- EGFR expression is induced by TCR engagement and sustained by cytokines, such as TSLP

Authors

Carlos M. Minutti, Sebastian Drube, Natalie Blair, ..., Padraic G. Fallon, Rick M. Maizels, Dietmar M. Zaiss

Correspondence

dietmar.zaiss@ed.ac.uk

In Brief

At the site of infection, Th2 cells secrete IL-13 upon exposure to IL-33. Minutti et al. now show that TCR-induced expression of the EGFR and its ligand amphiregulin was essential for IL-33-induced IL-13 secretion, revealing a mechanism whereby antigen-specific activation controls the innate effector function of Th2 cells.

Minutti et al., 2017, Immunity *47*, 710–722
October 17, 2017 © 2017 The Author(s). Published by Elsevier Inc.
https://doi.org/10.1016/j.immuni.2017.09.013

CellPress

CellPress

Immunity
Article

Epidermal Growth Factor Receptor Expression Licenses Type-2 Helper T Cells to Function in a T Cell Receptor-Independent Fashion

Carlos M. Minutti,[1] Sebastian Drube,[2] Natalie Blair,[1] Christian Schwartz,[3,4,5] Jame C. McCrae,[1] Andrew N. McKenzie,[6] Thomas Kamradt,[2] Michal Mokry,[7] Paul J. Coffer,[7] Maria Sibilia,[8] Alice J. Sijts,[9] Padraic G. Fallon,[3,4,5] Rick M. Maizels,[1] and Dietmar M. Zaiss[1,*]

[1]Institute of Immunology and Infection Research, University of Edinburgh, EH9 3FL Edinburgh, UK
[2]Institute of Immunology, Universitätsklinikum Jena, 07743 Jena, Germany
[3]Trinity Biomedical Sciences Institute, Trinity College Dublin, Dublin 2, Ireland
[4]National Children's Research Centre, Our Lady's Children's Hospital, Dublin12, Ireland
[5]Institute of Translational Medicine, Trinity College Dublin, Dublin 8, Ireland
[6]Medical Research Council (MRC) Laboratory of Molecular Biology, CB2 0QH Cambridge, UK
[7]Center for Molecular Medicine & Regenerative Medicine Center, University Medical Center Utrecht, 3584 CT Utrecht, the Netherlands
[8]Institute of Cancer Research, Department of Medicine I, Comprehensive Cancer Center, Medical University of Vienna, 1090 Vienna, Austria
[9]Department of Infectious Diseases & Immunology, Faculty Veterinary Medicine Utrecht, 3584 CL Utrecht, the Netherlands
*Correspondence: dietmar.zaiss@ed.ac.uk
https://doi.org/10.1016/j.immuni.2017.09.013

SUMMARY

Gastro-intestinal helminth infections trigger the release of interleukin-33 (IL-33), which induces type-2 helper T cells (Th2 cells) at the site of infection to produce IL-13, thereby contributing to host resistance in a T cell receptor (TCR)-independent manner. Here, we show that, as a prerequisite for IL-33-induced IL-13 secretion, Th2 cells required the expression of the epidermal growth factor receptor (EGFR) and of its ligand, amphiregulin, for the formation of a signaling complex between T1/ST2 (the IL-33R) and EGFR. This shared signaling complex allowed IL-33 to induce the EGFR-mediated activation of the MAP-kinase signaling pathway and consequently the expression of IL-13. Lack of EGFR expression on T cells abrogated IL-13 expression in infected tissues and impaired host resistance. EGFR expression on Th2 cells was TCR-signaling dependent, and therefore, our data reveal a mechanism by which antigen presentation controls the innate effector function of Th2 cells at the site of inflammation.

INTRODUCTION

Cytokines secreted at the site of infection crucially contribute to protective immunity against gastro-intestinal helminth infections (Anthony et al., 2007; Grencis, 2015). These cytokines induce diverse local effector functions that range from enhanced migration and turnover of intestinal epithelial cells (IECs), enhanced smooth muscle contractility, and local goblet cell hyperplasia to the induction of effector molecules such as the resistin-like molecules RELM alpha and beta or the *de novo* expression of the mucin Muc5ac, which has a direct detrimental effect on nematode vitality (Anthony et al., 2007; Hasnain et al., 2011). Which specific effector functions mediate pathogen-specific host resistance is dependent on the type of parasite, the physical location of the parasite within the gastro-intestinal tract, and the stage of infection (Anthony et al., 2007).

Cells at the site of infection that produce cytokines may be part of the innate immune system, such as type-2 innate lymphoid cells (ILC2), as well as of the adaptive immune system, such as pathogen-specific type-2 helper T cells (Th2 cells). ILC2, resident within mucosal tissues, can rapidly secrete cytokines upon exposure to interleukin-33 (IL-33) in an antigen-independent manner and, as demonstrated by adoptive transfer of *in-vitro*-expanded ILC2, can directly contribute to host resistance (Moro et al., 2010; Neill et al., 2010; Saenz et al., 2010). By contrast, pathogen-specific Th2 cells need first to be primed in an antigen-dependent manner, clonally expand, and migrate to the site of infection before they can contribute to nematode expulsion. Nevertheless, during gastro-intestinal helminth infections, Th2 cells rapidly outnumber ILC2 at the site of infection (Guo et al., 2015), and a number of publications suggest that these Th2 cells directly contribute to pathogen clearance (Anthony et al., 2006; Urban et al., 1992; Zaiss et al., 2006).

Although cytokine production by T cells is closely controlled by antigen-dependent T cell receptor (TCR) activation (Slifka and Whitton, 2000), it has recently been shown that Th2 cells can directly contribute to host resistance in a TCR-independent manner, by secreting the type-2 effector cytokine IL-13 upon exposure to IL-33 (Guo et al., 2015, 2009). This antigen-independent potential of Th2 cells to produce cytokines raises the question to which extent both antigen-dependent and -independent Th2 cell effector functions contribute to host resistance and whether these two functions are integrated.

We have shown before that activated effector CD4 T cells express the epidermal growth factor receptor (EGFR) (Zaiss et al.,

 © 2017 The Author(s). Published by Elsevier Inc.
This is an open access article under the CC BY license (http://creativecommons.org/licenses/by/4.0/).

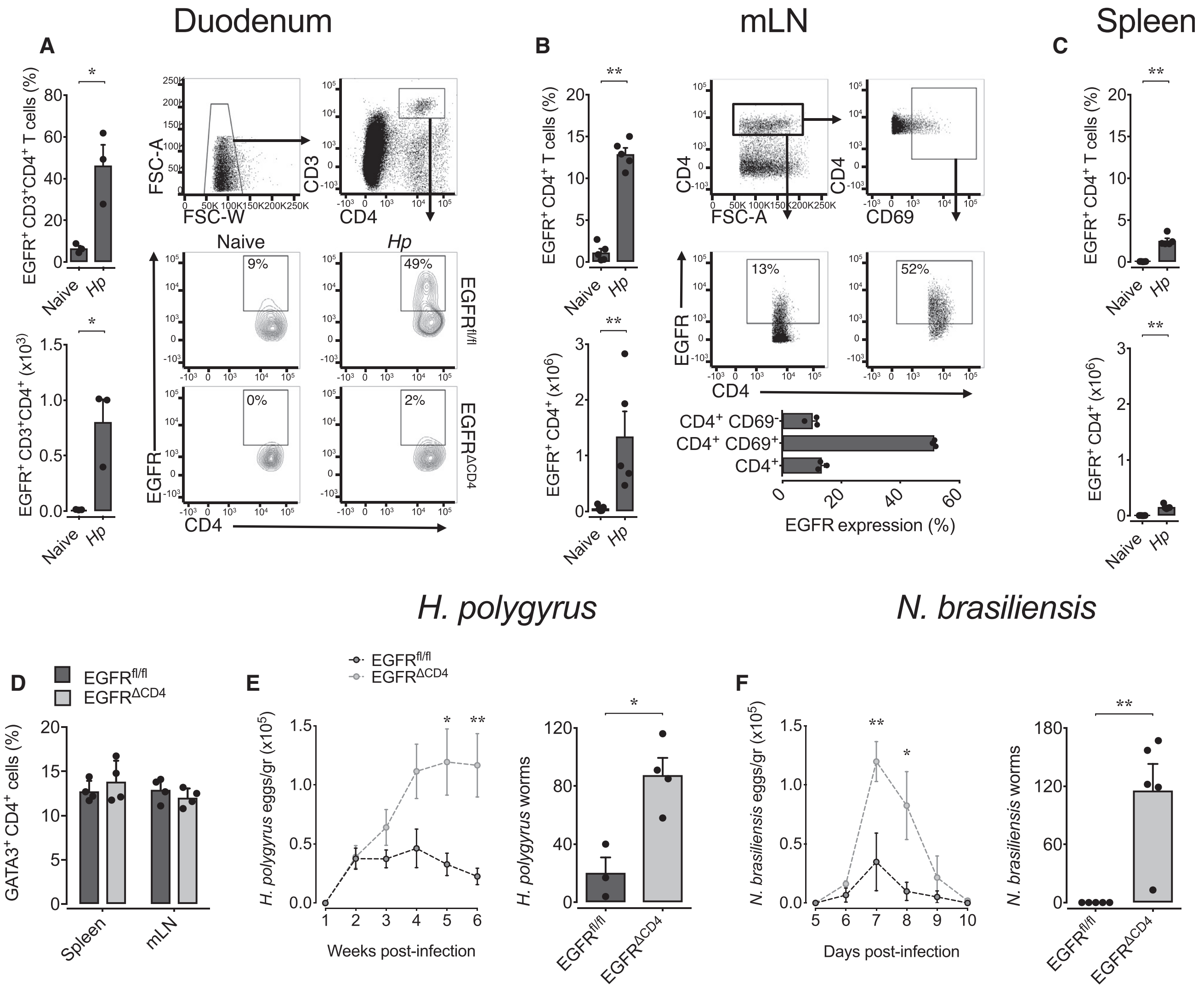

Figure 1. EGFR Expression by T Cells Contributes to Host Resistance against Gastro-intestinal Helminth Infections

(A–C) WT, *Egfr*$^{fl/fl}$, and *Egfr*$^{fl/fl}$*xCd4-cre* (EGFR$^{\Delta CD4}$) mice were infected with *H. polygyrus* or *N. brasiliensis* or left untreated. Percentage and absolute number of EGFR expressing CD4^{+} T helper cells in (A) duodena, (B) mLN, and (C) spleen, and EGFR expression on total CD4^{+} and CD69^{+} and CD69^{-} mLN-derived T cells from naive or *H. polygyrus*-infected mice was determined by flow cytometry analysis on day 14 post infection.

(D) Comparison of GATA3 expression on CD4^{+} T cells from mLN and spleen of *H. polygyrus*-infected *Egfr*$^{fl/fl}$ and EGFR$^{\Delta CD4}$ mice.

(E) Time-dependent egg load in feces and worm burden 4 weeks post infection with *H. polygyrus* (n = 4 mice).

(F) Time-dependent egg load in feces and worm burden 9 days post infection with *N. brasiliensis* (n = 3 mice).

All data are representative of at least two independent experiments (mean ± SEM); results for individual mice are shown as dots. See also Figures S1–S3.

2013). Here, we demonstrate that EGFR expression on Th2 cells was of critical importance for host resistance during gastro-intestinal helminth infections. Only following the induction of EGFR expression and the amphiregulin (AREG)-dependent formation of a functional signaling complex between the EGFR and T1/ST2 (the IL-33R) could activated Th2 cells secrete IL-13 in an antigen-independent way upon exposure to IL-33. EGFR expression by Th2 cells was induced by antigen-mediated activation. Thus, via the expression of the EGFR, TCR-mediated activation licenses Th2 cells to respond in an innate-like fashion, while at the same time controlling the overall immune response in an antigen-specific manner.

RESULTS

EGFR Expression by Activated Th2 Cells Contributes to Host Resistance

Since CD4^{+} effector T cells can express the EGFR (Liao et al., 2008; Zaiss et al., 2013), we explored whether Th2 cells might express the EGFR during helminth infections and whether EGFR expression contributes to host resistance. While we found negligible EGFR expression on CD4^{+} T cells from uninfected mice, EGFR expression was upregulated on CD4^{+} T cells derived from the duodena (Figure 1A) and mesenteric lymph nodes (mLN) (Figure 1B) of mice infected with the gastro-intestinal

helminth *Heligmosomoides polygyrus*. EGFR expression was restricted to recently activated $CD4^+$ T cells, as defined by the co-expression of the activation marker CD69 (Figure 1B). In contrast to mLN, the frequency of EGFR positive $CD4^+$ T cells in the spleen was lower (Figure 1C), although a comparable fraction of $CD4^+$ T cells expressed the Th2 transcription factor GATA3 in both lymphoid organs (Figure 1D).

To determine the physiological relevance of EGFR expression on T cells, we generated a mouse strain that lacks EGFR expression in T cells by crossing *Cd4-cre* mice onto an *Egfr*$^{fl/fl}$ background. Uninfected *Egfr*$^{fl/fl}$x*Cd4-cre* mice showed no signs of immune dysregulation, and T cell development appeared normal (Zaiss et al., 2013; Figure S1). However, *Egfr*$^{fl/fl}$x*Cd4-cre* mice infected with the gastro-intestinal helminth *H. polygyrus* showed significantly higher egg burdens and worm clearance was delayed significantly in comparison to infected wild-type (WT) control mice (Figure 1E). Similar results were obtained when *Egfr*$^{fl/fl}$x*Cd4-cre* mice were infected with *Nippostrongylus brasiliensis*, another gastro-intestinal nematode (Figure 1F).

Immunity to gastro-intestinal helminth infections is closely related to the expansion and function of regulatory T (Treg) cells (Allen and Maizels, 2011; Smith et al., 2016). Since Treg cell function is closely regulated by EGFR expression (Nosbaum et al., 2016; Okoye et al., 2014; Zaiss et al., 2013), we rationalized that Treg cell dysfunction may explain the enhanced susceptibility of *Egfr*$^{fl/fl}$x*Cd4-cre* mice to helminth infection. However, mice with a Treg cell-specific EGFR deficiency (*Foxp3-cre x Egfr*$^{fl/fl}$) cleared *H. polygyrus* as efficiently as WT control mice (Figure S2), suggesting that EGFR expression on Treg cells did not contribute to the enhanced susceptibility of *Egfr*$^{fl/fl}$*xCd4-cre* mice.

In order to determine the cause for the enhanced susceptibility of *Egfr*$^{fl/fl}$*xCd4-cre* mice to helminth infections, we analyzed the immune response of WT and *Egfr*$^{fl/fl}$*xCd4-cre* mice to *H. polygyrus* infection in more detail (Figure S3). We observed that percentages of $CD4^+$ T cells and their ability to produce IL-13 upon *in vitro* re-stimulation were comparable in both strains (Figure S3B). Pathogen-specific antibody titers within the serum and faeces were unaffected by a lack of EGFR expression on T cells (Figure S3C). Furthermore, we found similar frequencies of ILC2 in the mLN of infected *Egfr*$^{fl/fl}$*xCd4-cre* and WT control mice, and their capacity to produce type-2 cytokines upon IL-33 exposure was unaffected (Figure S3D), indicating that a lack of EGFR expression on T cells does not fundamentally affect ILC2 expansion and function. Furthermore, *in-vitro*-generated Th2 cells from *Egfr*$^{fl/fl}$*xCd4-cre* and WT mice expressed type-2-specific transcription factors GATA-3, c-MAF, and STAT-6 (Figure S3E), showed no major transcriptional deviations from each other (Figure S3E), and expanded similarly (Figure S3E).

From these data we conclude that EGFR expression on $CD4^+$ T cells is critical for Th2 cell-mediated host resistance to gastro-intestinal helminth infections. Nevertheless, a lack of EGFR expression does not induce a fundamental dysfunction of Th2 cells.

IL-13 Production at the Site of Infection Is Dependent on EGFR Expression by T Cells

One central component of host resistance against helminth infections is the expression of the effector cytokine IL-13 (Guo et al., 2015; McKenzie et al., 1998). *H. polygyrus* larvae are particularly sensitive to IL-13-induced effector mechanism from day 7 until day 9 post infection, when they leave the gut mucosa in order to reside in the intestinal lumen (Reynolds et al., 2012). We therefore determined cytokine production at the site of infection during this stage of infection. We found that *Il13* mRNA expression in the duodenum was significantly lower in infected *Egfr*$^{fl/fl}$*xCd4-cre* mice in comparison to WT mice (Figure 2A). This deficiency in cytokine expression was specific for IL-13, since the expression of *Il5* and *Il4* mRNA (Figure 2A) and the influx of T cells into the duodenum (Figure 2A) were unaffected. In contrast to the site of infection, *Il13* mRNA expression within the draining mLN was similar in both mouse strains (Figure S4A). Strikingly, the diminished message for *Il13* in the duodena of *Egfr*$^{fl/fl}$*xCd4-cre* mice directly correlated with a lack of Muc5ac expression (Figure 2B), an IL-13-induced mucin that directly affects gastro-intestinal nematode vitality (Hasnain et al., 2011).

In order to determine the cellular source of IL-13 at the site of infection, we injected brefeldin A into mice infected with *H. polygyrus*, at day 7 post infection. We harvested the duodenum 6 hr later and purified intestine-residential leukocytes in the presence of monensin. Since brefeldin A and monensin prevent the secretion of cytokines, this approach allowed us to directly reveal which cells expressed effector cytokines within the duodenum of infected mice. As shown in Figure 2C, $CD4^+$ T cells but not ILC2 expressed detectable amounts of IL-13 at this stage of infection. In addition, only $CD4^+$ T cells in infected WT but not in *Egfr*$^{fl/fl}$*xCd4-cre* mice expressed IL-13, while $CD4^+$ T cells from both mouse strains expressed similar amounts of IL-5 (Figures 2C, S4B, and S4C). Also, the numbers of $CD4^+$ T cells and ILC2 recovered from the infected duodena were comparable for both mouse strains (Figure S4D).

To confirm that a lack of IL-13 expression could be the cause of diminished worm clearance, we injected rIL-13 into *Egfr*$^{fl/fl}$*xCd4-cre* mice at days 6, 7, and 8 post infection. Supplementation of IL-13 at this stage of infection resulted in significantly lower egg counts and worm burdens in infected *Egfr*$^{fl/fl}$*xCd4-cre* mice, fully reverting their susceptible phenotype (Figure 2D). Furthermore, the transfer of $CD4^+$ T cells derived from infected WT but not from *Egfr*$^{fl/fl}$*xCd4-cre* donors restored host resistance of IL-13-deficient mice to *H. polygyrus* infections (Figure 2E). Similarly, transfer of purified $CD4^+$ T cells from *H. polygyrus*-infected WT donors into infected *Egfr*$^{fl/fl}$*xCd4-cre* mice significantly diminished egg output in *Egfr*$^{fl/fl}$*xCd4-cre* recipient mice (Figure 2F), whereas transfer of purified $CD4^+$ T cells from *Egfr*$^{fl/fl}$*xCd4-cre* donors did not.

Taken together, our data show that a lack of EGFR expression on T cells leads to diminished T cell-derived IL-13 expression in the duodenum at the time point when *H. polygyrus* worms establish themselves in the gut lumen. Diminished IL-13 expression at this stage of infection correlated with diminished Muc5ac expression and a defect in worm clearance, while application of recombinant IL-13 or transfer of $CD4^+$ T cells from infected WT mice was sufficient to restore worm clearance. Consequently, the lack of IL-13 expression by $CD4^+$ T cells at the site of infection is likely to be causative for the enhanced susceptibility of *Egfr*$^{fl/fl}$*xCd4-cre* mice to *H. polygyrus* infection.

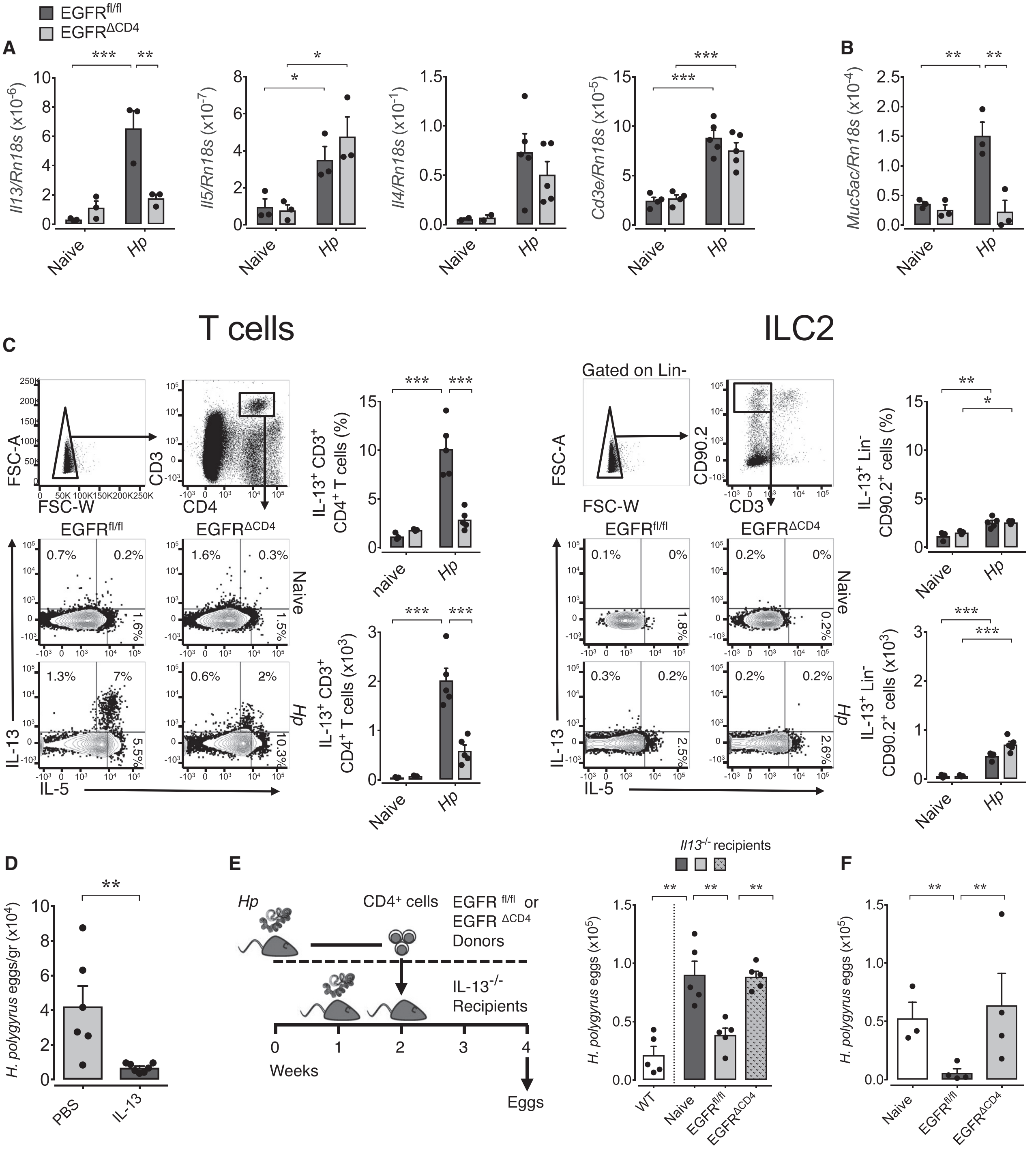

Figure 2. IL-13 Production at the Site of Infection Is Dependent on EGFR Expression by T Cells

WT, *Egfr*$^{fl/fl}$, and *Egfr*$^{fl/fl}$*xCd4-cre* (EGFR$^{\Delta CD4}$) mice were infected with *H. polygyrus* or left untreated.

(A–C) At day 8 post infection (A) *Il13*, *Il5, Il4, Cd3e*, and (B) *Muc5ac* mRNA was analyzed in the duodena by RT-PCR. (C) Mice were treated with BFA 6 hr prior to harvest, after which, single-cell suspensions from duodena of naive or infected mice were prepared and analyzed by flow cytometry: percentage and absolute number of IL-13-expressing T cells (left) and ILC2 (right).

(D) *H. polygyrus*-infected EGFR$^{\Delta CD4}$ mice received either PBS or IL-13 at days 6, 7, and 8 post infection, and egg counts were analyzed 2 weeks later.

(E) Flow cytometry-sorted mLN-derived CD4^{+} T cells were transferred from naive or *H. polygyrus*-infected *Egfr*$^{fl/fl}$ or EGFR$^{\Delta CD4}$ mice into infected (day 7 post infection) recipient *Il13*$^{-/-}$ mice, and eggs were counted in feces 2 weeks later; egg load on a WT strain is shown for reference.

(F) mLN-derived CD4^{+} T cells were transferred from naive or *H. polygyrus*-infected *Egfr*$^{fl/fl}$ or EGFR$^{\Delta CD4}$ mice into infected (day 7 post infection) recipient EGFR$^{\Delta CD4}$ mice, and eggs were counted in feces 2 weeks later.

All data are representative of at least two independent experiments (mean ± SEM); results for individual mice are shown as dots. See also Figure S4.

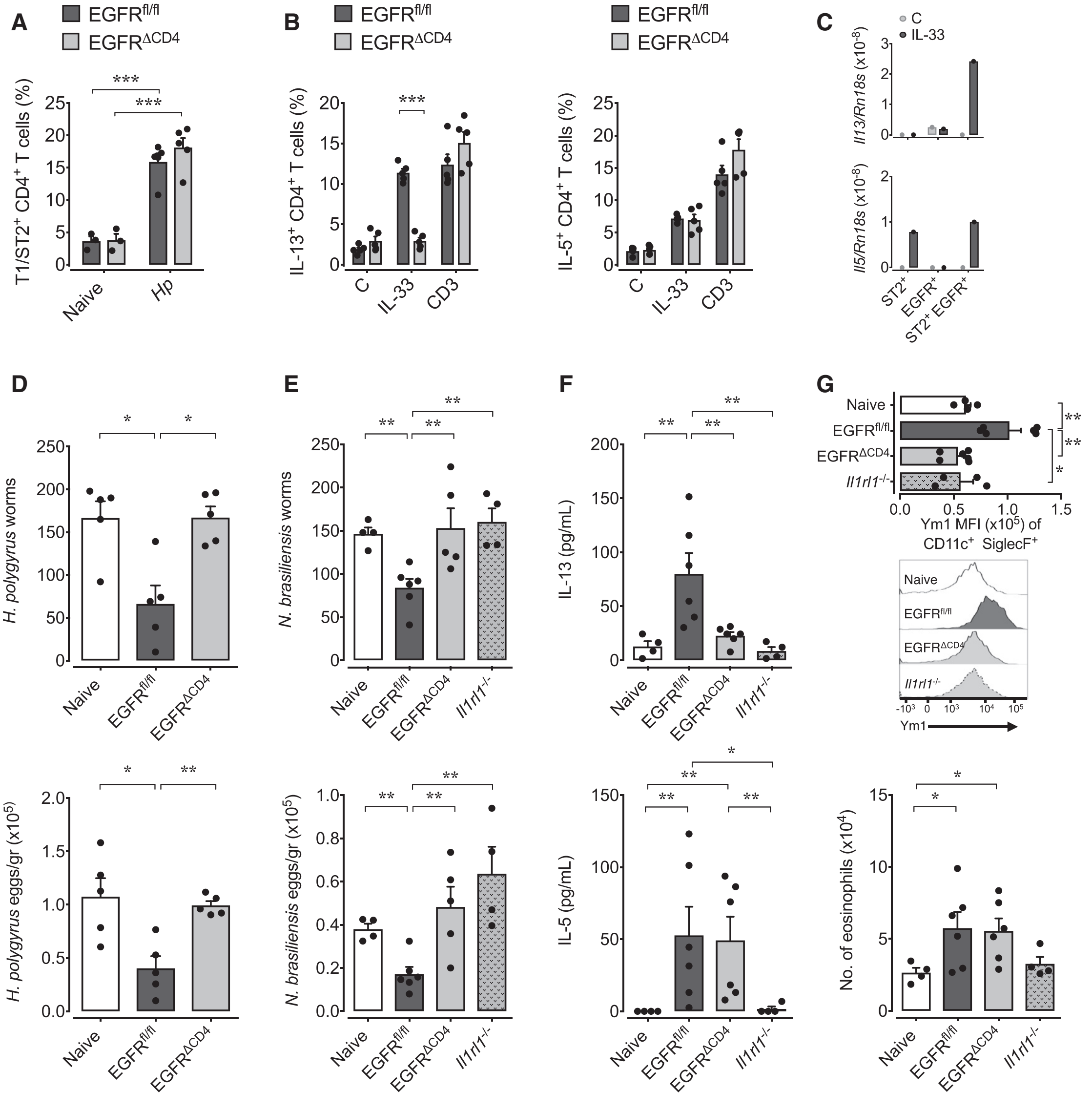

Figure 3. IL-33-Induced IL-13 Production by Th2 Cells Is Dependent on EGFR Expression

WT, *Egfr*$^{fl/fl}$, and *Egfr*$^{fl/fl}$*xCd4-cre* (EGFR$^{\Delta CD4}$) mice were infected with *H. polygyrus*, and on day 14 post infection, mLN were harvested.

(A) Comparison of T1/ST2 expression on mLN CD4^{+} T cells from *H. polygyrus*-infected *Egfr*$^{fl/fl}$ and EGFR$^{\Delta CD4}$ mice.

(B) mLN cells were stimulated with rIL-33, anti-CD3, or media only, and expression of IL-13 and IL-5 was determined by intra-cellular cytokine staining and flow cytometry analysis.

(C) mLN CD4^{+} T cells from *H. polygyrus*-infected WT mice were flow cytometry-sorted based on T1/ST2 and/or EGFR expression, and T cell populations were stimulated with rIL-33 for 4 hr. Subsequently, *Il13* and *Il5* mRNA was analyzed by RT-PCR.

(D) MHCII-deficient mice were infected with *H. polygyrus* and 7 days post infection received CD4^{+} T cells derived from mLN of naive or *H. polygyrus*-infected WT or EGFR$^{\Delta CD4}$ mice. Worm burden and egg counts were determined 2 weeks post infection.

(E–G) MHCII-deficient mice were infected with *N. brasiliensis* and simultaneously received CD4^{+} T cells from mLN of naive (WT) or *H. polygyrus*-infected WT, EGFR$^{\Delta CD4}$, or T1/ST2-deficient mice, collected 2 weeks after infection. At day 6 post transfer, worm burden and eggs counts (E) were determined, IL-13 and IL-5 (F) expression in BAL was evaluated by ELISA, and expression of Ym1 in alveolar macrophages and total number of eosinophils in the BAL (G) were analyzed by flow cytometry.

Data are representative of two independent experiments (mean ± SEM); results for individual mice are shown as dots.

IL-33-Induced IL-13 Production by Th2 Cells Is Dependent on EGFR Expression

IL-13 expression by Th2 cells can either be induced by TCR activation or in an antigen-independent way by exposure to IL-33 (Guo et al., 2009). In order to determine whether a lack of responsiveness to IL-33 by Th2 cells explained the diminished IL-13 expression in *Egfr*$^{fl/fl}$*xCd4-cre* mice, we determined IL-13 expression by CD4^{+} T cells derived from mice infected with *H. polygyrus ex vivo.* Despite similar expression of the IL-33 receptor T1/ST2 (Figure 3A), CD4^{+} T cells derived from *H. polygyrus*-infected *Egfr*$^{fl/fl}$*xCd4-cre* mice failed to express IL-13 upon exposure to IL-33. However, IL-33-induced IL-5

and IL-13 expression upon anti-CD3 stimulation was unaffected (Figure 3B). These data demonstrate that Th2 cells of *Egfr$^{fl/fl}$xCd4-cre* mice are capable of producing IL-13 upon antigen-specific stimulation but fail to produce IL-13 in response to IL-33.

To determine whether there might be a link between EGFR and T1/ST2 expression, we flow cytometry-sorted activated CD4$^+$ T cells (CD69$^+$) derived from the mLN of *H. polygyrus*-infected mice based on T1/ST2 and/or EGFR expression. As shown in Figure 3C, the induction of IL-13 in response to IL-33 exposure was restricted to the double-positive population, whereas IL-5 induction was dependent on T1/ST2 expression only. These findings demonstrate that T1/ST2-expressing Th2 cells require EGFR expression in order to produce IL-13 upon IL-33 exposure.

To address whether the previously described TCR-independent and IL-33-dependent capacity of Th2 cells to contribute to host resistance to helminth infections (Guo et al., 2015) requires EGFR expression, we transferred mLN-derived CD4 T cells from *H. polygyrus*-infected *Egfr$^{fl/fl}$xCd4-cre* or WT mice into *H. polygyrus*-infected major histocompatibility complex (MHC)-II-deficient mice, at day 7 post infection. While transfer of WT cells significantly diminished worm burden, transfer of cells derived from infected *Egfr$^{fl/fl}$xCd4-cre* mice did not (Figure 3D). Thus, the innate capacity of Th2 cells to contribute to worm expulsion is directly dependent on EGFR expression.

Furthermore, we decided to determine how the expression of EGFR on Th2 cells influences their innate-like cytokine expression within secondary inflamed tissues. To this end, we transferred purified CD4$^+$ T cell derived from the mLN of *H. polygyrus*-infected mice into *N. brasiliensis*-infected MHC-II-deficient mice. While *H. polygyrus* exclusively infects the gastro-intestinal tract, *N. brasiliensis* larvae migrate through the lung on route to intestine colonization, leaving behind a strong inflammatory environment. Transfer of purified CD4$^+$ T cells derived from *H. polygyrus*-infected WT mice into *N. brasiliensis*-infected MHC-II-deficient mice led to a significant reduction in worm burdens (Figure 3E). In contrast, transfer of CD4$^+$ T cells from infected *Egfr$^{fl/fl}$xCd4-cre* or T1/ST2-deficient mice did not (Figure 3E).

Of note, *N. brasiliensis*-infected MHC-II-deficient mice that had received CD4$^+$ T cells derived from *H. polygyrus*-infected WT mice showed significantly elevated IL-13 and IL-5 expression in the bronchoalveolar lavage (BAL) (Figure 3F). In contrast, MHC-II-deficient mice that had received CD4$^+$ T cells derived from *Egfr$^{fl/fl}$xCd4-cre* mice showed elevated IL-5 but lower IL-13 expression (Figure 3F). MHC-II-deficient mice that had received CD4$^+$ T cells derived from *H. polygyrus*-infected T1/ST2-deficient mice lacked detectable quantities of either cytokine (Figure 3F). Consistent with these cytokine expression profiles, alveolar macrophages from mice that received WT CD4$^+$ T cells showed enhanced expression of Ym1 (Figure 3G), a marker of alternative activation of macrophages that can be induced through IL-13 signaling via the IL-4Rα chain. However, mice that received either T1/ST2-deficient or EGFR-deficient CD4$^+$ T cells failed to induce alternative activation of alveolar macrophages (Figure 3G). In contrast to macrophage skewing, expansion of eosinophils in the BAL of *N. brasiliensis*-infected MHC-II-gene-deficient mice, a process dependent on IL-5 expression, was enhanced in mice that received WT or EGFR-deficient CD4$^+$ T cells but was absent in mice that received T1/ST2-deficient CD4$^+$ T cells (Figure 3G).

Taken together, our data demonstrate that EGFR expression by Th2 cells regulates their antigen-independent cytokine expression in response to IL-33.

Induced EGFR Expression Licenses Th2 Cells to Express IL-13 in an Innate-like Way

Next, we wanted to determine which factors influence EGFR expression on Th2 cells. In contrast to Th2 cells derived from the duodenum and mLN of infected mice, Th2 cells derived from the spleen of *H. polygyrus*-infected mice had lower EGFR expression (Figure 1A–1C). We therefore used splenic CD4$^+$ T cells from *H. polygyrus*-infected mice to determine which stimuli induce EGFR expression on CD4$^+$ T cells. We stimulated these cells overnight with either cytokines or via the TCR, using antigen-specific stimulation or activating anti-CD3 antibody. Stimulation through the TCR induced EGFR expression in CD4$^+$ T cells (Figure 4A). In addition, as described before (Liao et al., 2008), exposure to cytokines that induce STAT5 signaling, such as IL-2, IL-7, or TSLP, also induced EGFR expression in CD4$^+$ T cells; however, exposure to IL-33 did not (Figure 4A). In accordance with EGFR expression, overnight exposure to TSLP, a cytokine expressed at sites of helminth infection (Taylor et al., 2009), restored the capability of WT but not *Egfr$^{fl/fl}$xCd4-cre* splenic T cells from *H. polygyrus*-infected mice to produce IL-13 in response to IL-33 (Figure 4A), demonstrating that induced EGFR expression licenses Th2 cells to produce IL-13 upon exposure to IL-33.

In addition to the induction of EGFR expression, we also wanted to know for how long Th2 cells remain responsive to IL-33 and whether this sensitivity would correlate with EGFR expression. While T1/ST2 expression on activated Th2 cells is known to be stable (Löhning et al., 1998), the duration of EGFR expression on activated Th2 cells remains unknown. To address EGFR stability, we transferred activated Th2 cells from *H. polygyrus*-infected WT mice into naive mice (Figure 4B) or, alternatively, induced worm clearance by administering a deworming drug (Figure 4C). As shown in Figure 4, EGFR expression on activated Th2 cells dropped rapidly upon transfer from infected Ly5.2 mice into uninfected Ly5.1 mice or upon drug-mediated worm expulsion. This drop in EGFR expression directly correlated with a loss of capacity of Th2 cells to produce IL-13 upon IL-33 exposure (Figures 4B and 4C). In contrast, the capacity of these cells to produce IL-5 upon exposure to IL-33 remained unimpaired, which directly correlated with the unaffected T1/ST2 expression on Th2 cells (Figures 4B and 4C).

Taken together, these data demonstrate that EGFR expression on Th2 cells is only transient and rapidly lost in the absence of inflammation.

Antigen-Independent Function of Th2 Cells Requires Antigen-Induced EGFR Expression

Our data show that cytokines, such as TSLP, can induce EGFR expression in CD4$^+$ T cells derived from *H. polygyrus*-infected mice. Therefore, we wanted to determine whether, under physiological conditions, EGFR expression on Th2 cells can be driven by cytokine-induced bystander activation or at one point has to be controlled by antigen presentation. To this end, we compared

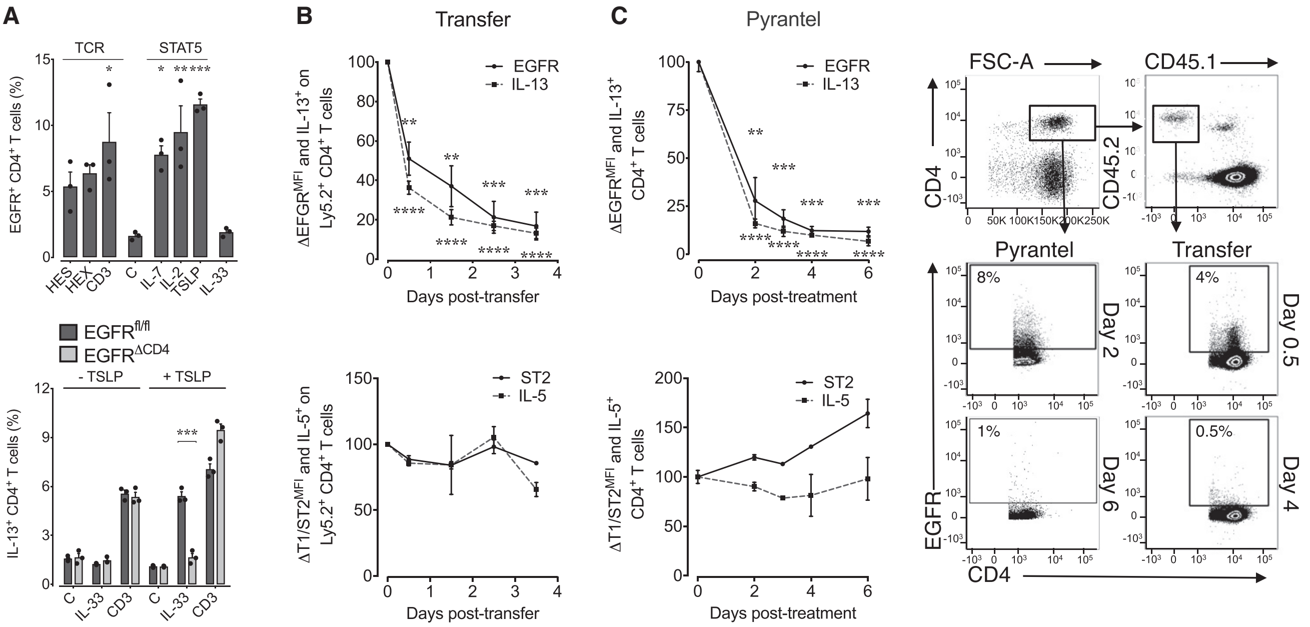

Figure 4. Transient EGFR Expression Licenses Th2 Cells to Express IL-13 in Response to IL-33

(A) Splenocytes were isolated from *H. polygyrus*-infected WT and *Egfr$^{fl/fl}$xCd4-cre* (EGFR$^{\Delta CD4}$) mice, and expression of EGFR on T cells was determined by flow cytometry analysis after overnight incubation with *H. polygyrus* excretory-secretory products (HES), adult worm extract (HEX), anti-CD3, vehicle (C), rIL-7, rIL-2, rTSLP, or rIL-33 (upper). Alternatively (lower), splenocytes were stimulated overnight with rTSLP, and subsequently, IL-13 expression by T cells was analyzed by intra-cellular cytokine staining and flow cytometry analysis following re-stimulation with rIL-33 or anti-CD3 antibody.

(B) Ly5.2$^+$ WT mice were infected with *H. polygyrus*, and 2 weeks after infection, mLN cells were transferred into naive Ly5.1$^+$ hosts. Spleens of recipient mice were harvested after 0–3.5 days post transfer, and the expression of EGFR (upper) and T1/ST2 (lower) on transferred Ly5.2$^+$ CD4$^+$ T cells was determined by flow cytometry analysis. Expression of IL-13 (upper) and IL-5 (lower) upon exposure to rIL-33 was determined by intra-cellular cytokine expression and flow cytometry analysis (n = 3 mice).

(C) WT *H. polygyrus*-infected mice were treated with pyrantel embonate, and mLN were harvested after 0–6 days post treatment. EGFR (upper) and T1/ST2 (lower) expression on CD4$^+$ T cells was determined by flow cytometry, and expression of IL-13 (upper) and IL-5 (lower) upon exposure to IL-33 was determined by intra-cellular cytokine staining and flow cytometry analysis (n = 3 mice).

Data are representative of at least two independent experiments (mean ± SEM); results for individual mice are shown as dots. Data in (B) and (C) are presented as percentage of expression on day 0.

EGFR expression on mLN-derived CD4$^+$ T cells from *H. polygyrus*-infected mice or memory CD4$^+$ T cells from drug-treated mice. Figure 5A shows that, whereas recently activated CD4$^+$ T cells increase EGFR expression, memory cells do not. Thus, we sorted memory cells (CD45RB^{low}CD25$^-$CD44^{high}) and activated Th2 cells (CD69$^+$ST2$^+$) based on EGFR expression (Figure 5B) and either exposed these cell populations directly to IL-33 or cultured memory and activated EGFR$^-$ CD4$^+$ T cells in the presence of CD3-stimulating antibodies or TSLP overnight. We observed that EGFR-expressing activated Th2 cells readily expressed IL-13 upon IL-33 exposure, while memory CD4$^+$ T cells did not (Figure 5C). Furthermore, we observed that TCR-mediated activation and TSLP stimulation could induce EGFR expression in the activated cell population that were isolated based on a lack of EGFR expression; however, CD4$^+$ T cells within the memory population expressed EGFR only upon TCR-mediated activation, while TSLP alone was insufficient to induce EGFR expression (Figure 5D). Furthermore, the capability of the different sorted cell populations to express IL-13 in response to IL-33 was directly correlated to EGFR expression (Figure 5E).

In order to address the *in vivo* relevance of antigen presentation for the re-activation of memory CD4$^+$ T cells, we transferred CD4$^+$ T cells into MHC-II-deficient mice. Transferred cells were either derived from infected mice or from mice that had been drug-treated 2 weeks prior to transfer. While CD4$^+$ T cells from infected mice significantly diminished worm burden, transfer of CD4$^+$ T cells from drug-treated mice did not (Figure S5A). However, transfer of CD4$^+$ T cells from dewormed WT mice was sufficient to induce worm expulsion in infected *Egfr$^{fl/fl}$xCd4-cre* recipients (Figure S5B), suggesting that antigen-specific re-activation enabled the transferred resting Th2 cells to contribute to worm expulsion.

In order to exclude possible variations in numbers of transferred T cells, we modified the previous experimental approach and immunized WT and *Egfr$^{fl/fl}$xCd4-cre* mice with ovalbumin (OVA) and subsequently repeatedly challenged these mice with OVA intra-nasally. We then harvested thoracic lymph nodes and sorted activated T cells immediately after challenge or memory CD4$^+$ T cells 4 weeks post challenge, and equal number of memory or activated CD4$^+$ T cells were transferred into *H. polygyrus*-infected *Egfr$^{fl/fl}$xCd4-cre* recipient mice. As shown in Figure 5F, transfer of activated WT but not *Egfr$^{fl/fl}$xCd4-cre* CD4$^+$ T cells induced worm expulsion. Memory CD4$^+$ T cells, however, were not able to provide protection unless recipient mice were immunized with OVA (Figure 5G), whereby worm

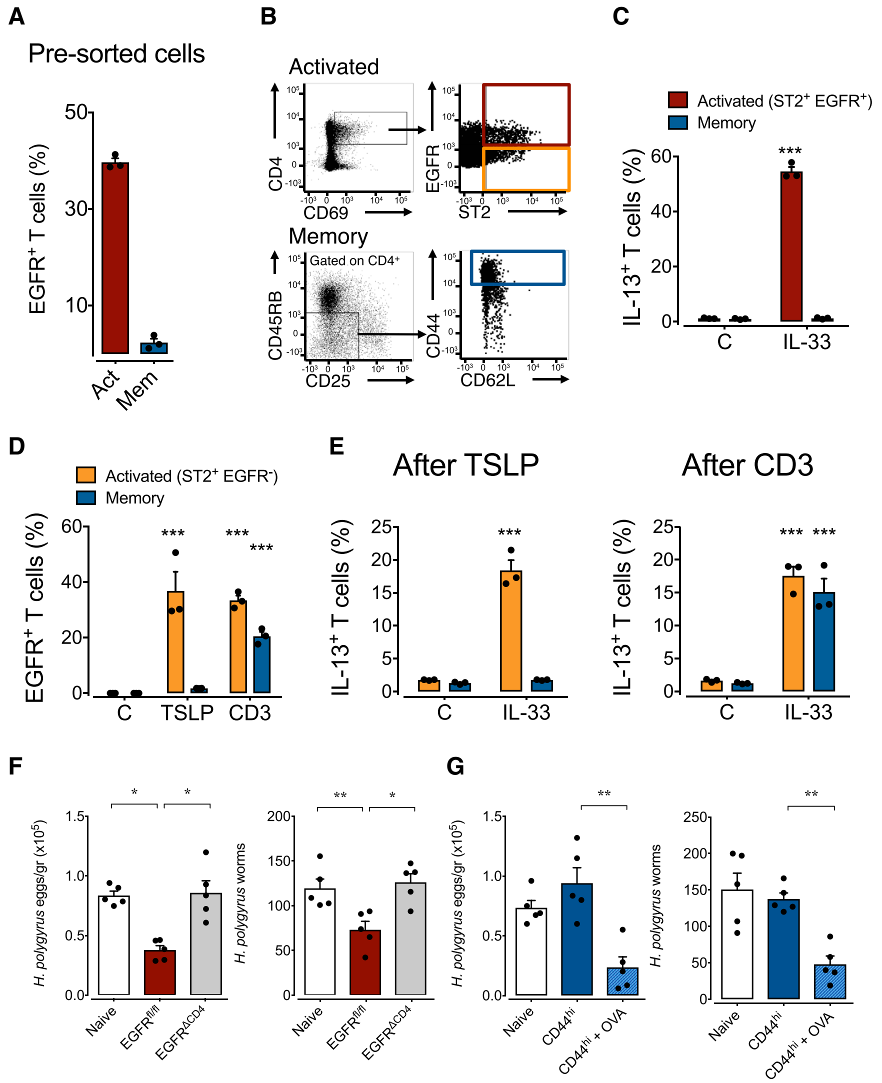

Figure 5. EGFR Expression on Th2 Cells Is Induced by TCR Activation and Maintained by Cytokines

(A–E) WT *H. polygyrus*-infected mice were either treated with pyrantel embonate or left untreated; mLN were collected 14 days after treatment.

(A) EGFR expression on activated ($CD69^+$, red bars) or memory ($CD25^-CD45RB^{low}CD44^{hi}$, blue bars) $CD4^+$ T cells was analyzed by flow cytometry.

(B) Activated $CD4^+$ T cells were flow cytometry-sorted from infected mice into $ST2^+$ $EGFR^+$ (red) and $ST2^+$ $EGFR^-$ (orange) populations. Memory $CD4^+$ T cells ($CD25^-CD45RB^{low}CD44^{hi}$) were flow cytometry-sorted from dewormed mice.

(C) After sorting, activated $EGFR^+$ and memory cells were stimulated with rIL-33 or media only, and expression of IL-13 was determined by flow cytometry analysis.

(D–E) Activated $EGFR^-$ and memory cells were cultured overnight with rTSLP or anti-CD3 antibodies, and expression of EGFR on $CD4^+$ T cells was determined by flow cytometry analysis (D) or were re-stimulated with rIL-33 and expression of IL-13 determined by intra-cellular cytokine staining and flow cytometry analysis (E).

(F–G) *Egfr^fl/fl^xCd4-cre* ($EGFR^{\Delta CD4}$) mice were infected with *H. polygyrus* and received either flow cytometry-sorted activated CD4 T cells (red) (F) or flow cytometry-sorted naive or memory T cells (blue) (G) from OVA-challenged mice. Worm burden and egg counts in feces were determined 2 weeks post transfer.

Data in (A)–(E) are representative of at least two independent experiments (mean ± SEM); results for individual mice are shown as dots. See also Figure S5.

expulsion directly correlated with the presence of EGFR-expressing $CD4^+$ T cells within the duodenum (Figure S5C).

Taken together, these experiments show that only antigen-specific activation of Th2 cells induces EGFR expression, while cytokines maintain EGFR expression in recently activated Th2 cells.

T1/ST2 and EGFR Form a Common Signaling Complex on Th2 Cells

Finally, we wanted to determine the mechanism by which EGFR expression controls IL-33-induced cytokine expression. IL-13 expression is critically dependent on ERK activation (Pahl et al., 2002), and because MAP-kinase signaling is one of the principal pathways induced by EGFR activation, we hypothesized that EGFR-mediated activation of MAP-kinase signaling may facilitate IL-33 to induce IL-13 expression in Th2 cells. In accordance with this hypothesis, IL-33 failed to induce ERK phosphorylation in EGFR-deficient $CD4^+$ T cells (Figure 6A), and treatment of T cells derived from *H. polygyrus*-infected WT mice with MEK inhibitors, which interfere with the MAP-kinase signaling pathway, phenocopied *Egfr^fl/fl^xCd4-cre* T cells (Figure S6A). IL-33-induced IL-5 and IL-4 expression (Figure S6B) as well as IκBα degradation (Figure S6B) were unaffected by a lack of EGFR expression. This finding is in accordance with the fact that IL-5 expression is dependent on p38 but not on ERK activation (Endo et al., 2015). Accordingly, IL-33-induced IL-5 expression was unaffected by MEK inhibitor treatment (Figure S6C). These data demonstrate that specifically IL-33-induced ERK activation is EGFR dependent, while other signaling pathways downstream of T1/ST2 are not.

In order to induce EGFR signaling upon IL-33 exposure, T1/ST2 could either form an active signaling complex together with the EGFR, or T1/ST2 could induce the expression or release of EGFR ligands that then indirectly activate the EGFR.

To address these possibilities, we first exposed $CD4^+$ T cells derived from *H. polygyrus*-infected mice with IL-33 in the presence of the EGFR inhibitor gefitinib and the pan-metalloprotease inhibitor marimastat, which disrupts the release of newly expressed EGFR ligands from the cell surface. EGFR inhibition prevented IL-33-induced IL-13 production and ERK phosphorylation (Figures 6B and 6C), while the inhibition of EGFR transactivation by marimastat had no influence on IL-33-induced

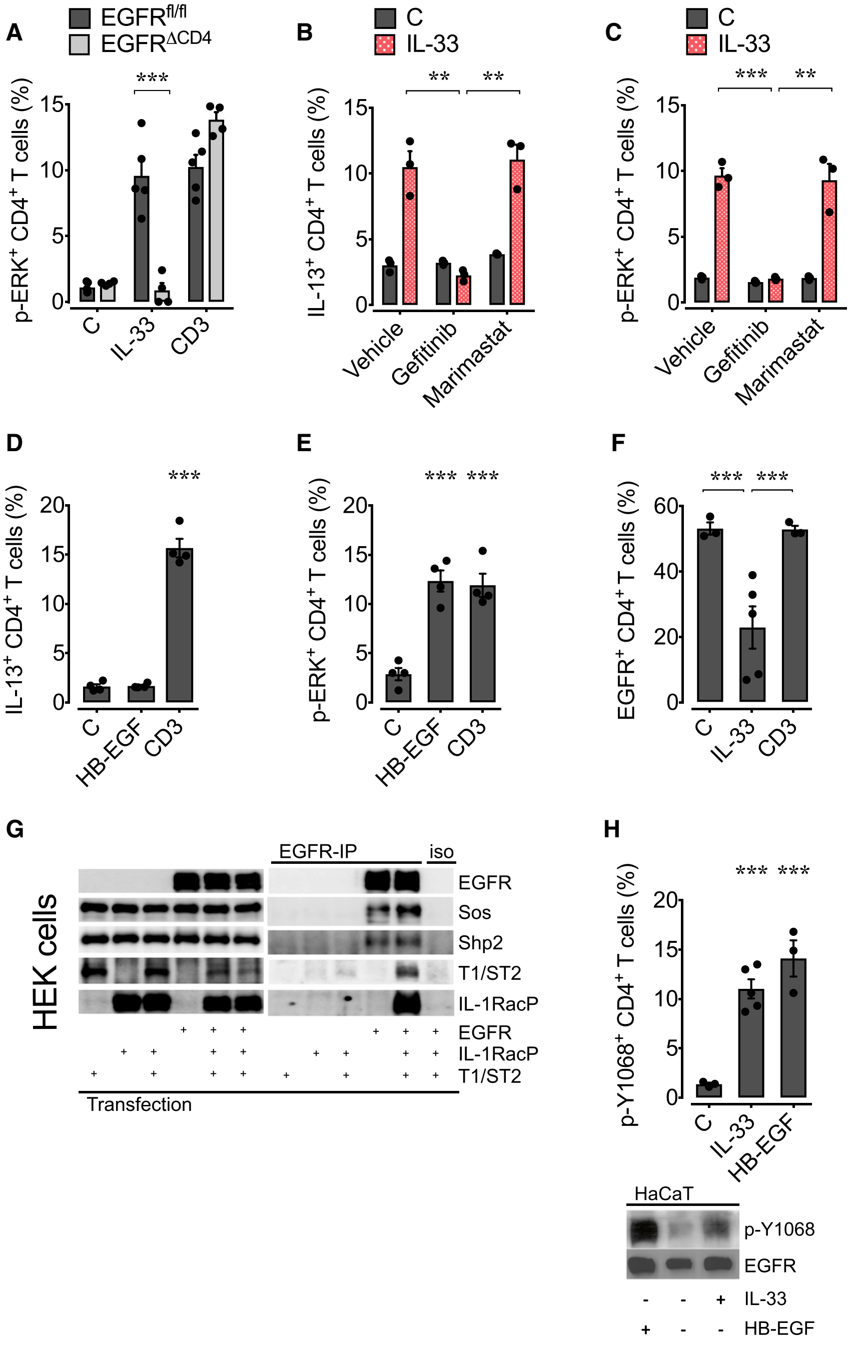

Figure 6. IL-33-Induced IL-13 Production by Th2 Cells Is Dependent on a Signaling Complex between T1/ST2 and EGFR

WT and *Egfr*$^{fl/fl}$*xCd4-cre* (EGFR$^{\Delta CD4}$) mice were infected with *H. polygyrus* larvae, and on day 14 post infection, mLN were harvested.

(A–C) Cells were stimulated with rIL-33, anti-CD3, or media in the presence of gefitinib, marimastat, or vehicle, and p-ERK (A and C) or IL-13 (B) expression was determined by intra-cellular staining and flow cytometry analysis.

(D and E) Cells were stimulated with rHB-EGF, anti-CD3, or media, and IL-13 (D) and p-ERK (E) expression was determined by intra-cellular staining and flow cytometry analysis.

(F) EGFR expression on stimulated mLN WT CD4$^+$ T cells in the presence of monensin was analyzed by flow cytometry.

(G) HEK293T cells were transfected as indicated with T1/ST2, the IL-1RacP, or the EGFR alone or in combination. Subsequently, the cells lysates were analyzed for the expression of the transfected proteins (input, left panel). The same lysates were also subjected to an EGFR-specific immunoprecipitation (EGFR-IP, right panel) or were treated with the isotype control (iso, right panel). Precipitates were analyzed by immunoblot.

(H) mLN (upper) or HaCaT cells (lower) were stimulated with rIL-33, rHB-EGF, or media, and the EGFR phosphorylation at position Y1068 was determined by intra-cellular staining and flow cytometry analysis (upper) or immunoblot (lower).

All data are representative of at least two independent experiments (mean ± SEM); results for individual mice are shown as dots. See also Figure S6.

IL-13 expression nor on ERK phosphorylation (Figures 6B and 6C). Furthermore, we observed that the high-affinity EGFR ligand HB-EGF was not able to induce IL-13 expression by CD4$^+$ T cells despite inducing ERK activation in these cells (Figures 6D and 6E), indicating that IL-33-induced IL-13 expression is not mediated by the release of EGFR ligands.

To address whether both receptors may physically interact, we studied the internalization of the EGFR on IL-33-treated CD4$^+$ T cells derived from *H. polygyrus*-infected mice in the presence of monensin. Monensin allows for activation-induced internalization of trans-membrane receptors but prevents re-circulation of these receptors back to the cell surface. As shown in Figure 6F, IL-33 induced the internalization of the EGFR. This activation-induced internalization was specific for IL-33 exposure and not observed upon TCR-mediated stimulation (Figure 6F). Additionally, EGFR co-immunoprecipitated with T1/ST2 and its associated signaling molecules, such as SOS and Shp-2, when co-expressed in HEK cells (Figure 6G). Similarly, EGFR co-immunoprecipitated with T1/ST2 from IL-33-treated CD4$^+$ T cells derived from *H. polygyrus*-infected WT but not from *Egfr*$^{fl/fl}$*xCd4-cre* mice (Figure S6D), further suggesting that both receptors may form a shared complex.

EGFR-induced activation of the MAP-kinase signaling pathway is mediated via the phosphorylation of Tyr-1068. Thus, we wanted to test whether IL-33 may induce Tyr-1068 of the EGFR. As shown in Figure 6H, IL-33 induced the rapid phosphorylation of Tyr-1068 in CD4$^+$ T cells derived from *H. polygyrus*-infected mice (Figure 6H) as well as in HaCat cells (Figure 6H).

Based on these data, we conclude that, similar to the reported interaction between T1/ST2 and c-kit on mast cells (Drube et al.,

2010), T1/ST2 forms an active signaling complex with the EGFR on Th2 cells, which enables IL-33 to activate the MAP-kinase signaling pathway and induce ERK phosphorylation and consequently IL-13 expression.

Formation of a Common Signaling Complex Is Dependent on Th2 Cell-Derived Amphiregulin

Another important type-2 cytokine and EGF-like growth factor is AREG (Zaiss et al., 2015). Similar to that of EGFR (Figure 4A), AREG expression in Th2 cells is also induced by TCR and TSLP, but not IL-33 (Figure S7A). We have shown that Th2 cell-derived AREG enhances the clearance of the gastro-intestinal helminth *Trichuris muris* (Zaiss et al., 2006), and we therefore asked whether this EGF-like growth factor contributes to IL-33-induced IL-13 production. Using *Areg*$^{-/-}$ mice, we found that IL-33 could not induce IL-13 expression and ERK activation in CD4^{+} T cells derived from the mLN of *H. polygyrus*-infected mice (Figure 7A and 7B), although EGFR expression on Th2 cells and the fraction of CD4^{+} T cells expressing the EGFR were similar in WT as in *Areg*$^{-/-}$ mice (Figure S7B). Also, IL-33-induced IL-5 expression was unaffected (Figure S7C). Moreover, transfer of CD4^{+} T cells derived from *H. polygyrus*-infected *Areg*$^{-/-}$ mice could not induce worm expulsion in MHC-II-deficient mice (Figure 7C), suggesting that Th2 cell-derived AREG contributes to worm expulsion in an autocrine fashion, enabling Th2 cells to function in an innate-like manner.

Mechanistically, we found that in activated Th2 cells derived from *H. polygyrus*-infected *Areg*$^{-/-}$ mice, IL-33 could only induce phosphorylation of Tyr-1068 of the EGFR in the presence of rAREG and thus ERK phosphorylation and IL-13 expression (Figure 7D–7F). Furthermore, in CD4^{+} T cells derived from *H. polygyrus*-infected WT mice, position Tyr-992 of the EGFR is constitutively phosphorylated, while this phosphorylation was missing in *Areg*$^{-/-}$. Nevertheless, rAREG could restore this phosphorylation (Figure 7G). Co-expression and co-immunoprecipitation of an EGFR-Y992F mutant with T1/ST2 in HEK cells further demonstrated that activation via Tyr-992 of the EGFR is essential for the formation of a hetero-complex between T1/ST2 and the EGFR (Figure 7H). This finding was further supported by the fact that EGFR co-immunoprecipitation (Figure S7D) and EGFR was co-internalized in the presence of monensin was enhanced in response to IL-33 treatment on CD4^{+} T cells derived from *H. polygyrus*-infected WT mice but not on CD4^{+} T cells derived from *H. polygyrus*-infected *Areg*$^{-/-}$ mice (Figure 7I). Taken together, these data reveal that the AREG-induced Tyr-992 phosphorylation of the EGFR on Th2 cells allows for the formation of an active signaling complex between the EGFR and T1/ST2, essential for IL-33-induced IL-13 expression (Figure S7E).

In conclusion, our data demonstrate that the innate-like effector function of Th2 cells at the site of infection is controlled by EGFR and AREG expression. In order to initiate their expression, Th2 cells require antigen-dependent activation, while their expression on activated Th2 cells is sustained by cytokine-induced signaling. In this way, TCR-mediated T cell activation controls the innate effector function of Th2 cells via EGFR expression.

DISCUSSION

Several groups have shown before that Th2 cells contribute to worm expulsion at the site of infection (Anthony et al., 2006; Guo et al., 2015; Urban et al., 1992; Zaiss et al., 2006). These findings raise the question of how Th2 cells are activated at the site of infection. A number of cell types, such as macrophages, endothelial cells, and ILC2, express MHC-II molecules and could therefore potentially present pathogen-derived antigens to Th2 cells. One well-established example for such an antigen-specific interaction at the site of infection is the MHC-II-mediated interaction between ILC2 and Th2 cells (Oliphant et al., 2014). Nevertheless, antigen presentation at the site of infection is inherently less well organized than within the site-draining lymph nodes. In addition, a number of helminths not only fundamentally change protein expression during development but also have been shown to secrete products that directly interfere with antigen presentation (Carvalho et al., 2009), suggesting that local immune escape mechanisms could limit the effectiveness of local antigen-based cytokine expression. The group of W. Paul demonstrated that Th2 cells, similar to ILC2, can function in an antigen-independent way, by secreting the effector cytokine IL-13 upon exposure to IL-33 (Guo et al., 2009). IL-33 release, however, is found under a number of different inflammatory conditions. Thus, this TCR-independent way of cytokine expression by Th2 cells raised the question of how cytokine expression by Th2 cells might be controlled to prevent aberrant expression of IL-13, which can cause immune pathology and fibrosis (Wynn and Ramalingam, 2012). Our data reveal an unexpected mechanism by which TCR-independent cytokine expression by Th2 cells is regulated. Th2 cells are primed and expand in a clonal, antigen-dependent manner. Once activated, Th2 cells upregulate EGFR expression and express AREG, which allows them to secrete the effector cytokine IL-13 following exposure to IL-33. Thus, in effect, via this EGFR-mediated licensing of Th2 cells, we observed a functional merger of the innate and adaptive immune responses at the site of infection in order to reach a sufficient mass at critical time points during infection. In this process, the involvement of antigen-specific Th2 cells allows for the rapid expansion of cells that can respond to infection independent of local antigen presentation, while at the same time these cells remain in their functional state closely controlled by antigen presentation, thereby assuring for optimal host resistance while preventing immunopathology.

Our finding that the interaction between T1/ST2 and the EGFR is further controlled by the type-2 cytokine and EGF-like growth factor AREG dovetails with our former findings demonstrating that AREG contributes to the efficient clearance of *Trichuris muris* infection (Zaiss et al., 2006). In that publication, we showed that Th2-derived AREG contributed to helminth expulsion by enhancing the proliferation of the IEC layer at the site of infection. These findings appeared to be in conflict with the findings by the group of Richard Grencis, who had shown that the enhanced proliferation of the IEC layer was IL-13 induced (Cliffe et al., 2005). Our current data now show that Th2 cell-expressed AREG in an autocrine fashion facilitates the IL-33-induced expression of IL-13 at the site of infection. Thus, it is tempting to speculate that also during *T. muris* infections, AREG may contribute to host resistance by licensing activated Th2 cells to

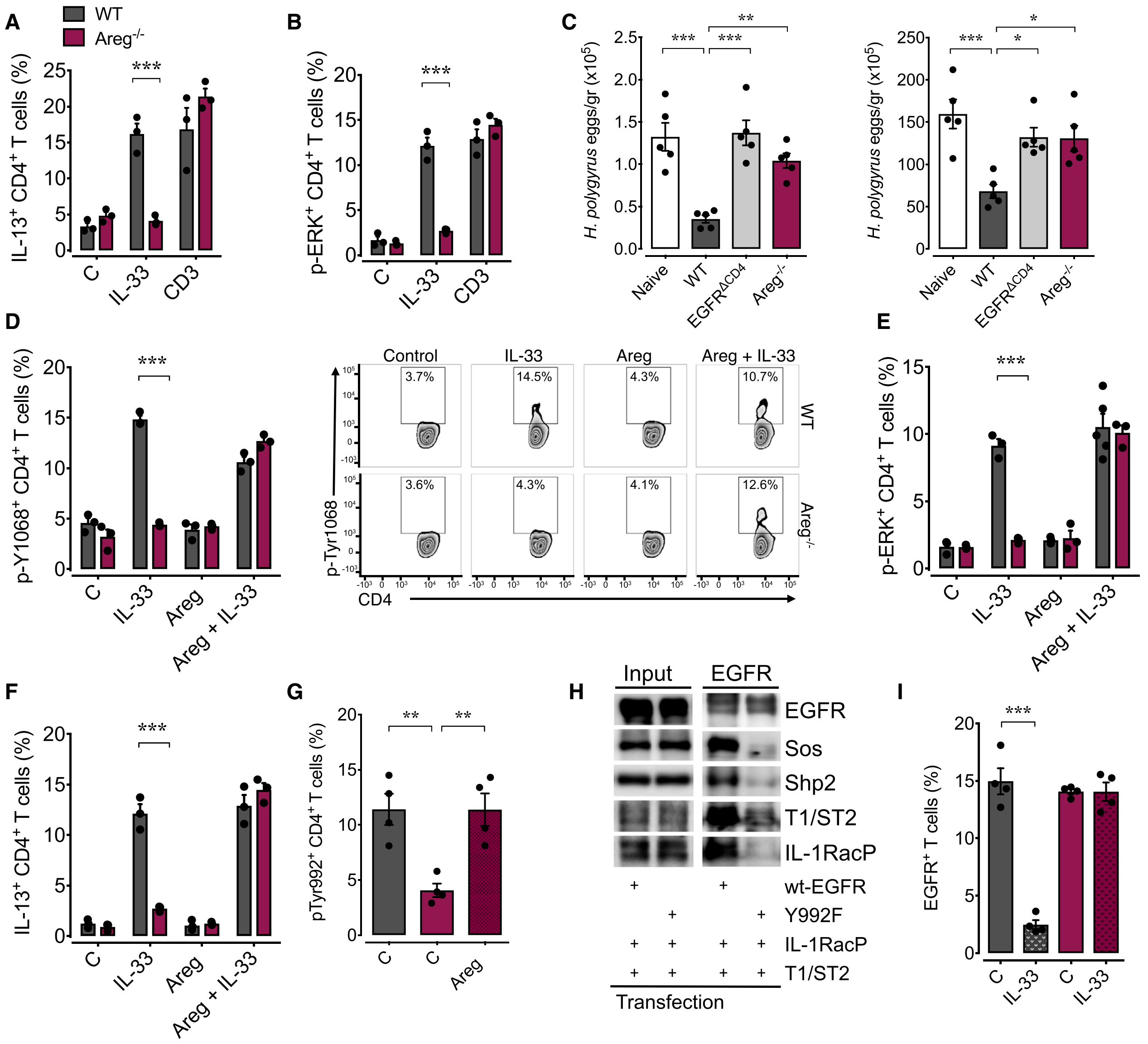

Figure 7. AREG Induces EGFR Phosphorylation at Tyr-992, which Allows for the Interaction between T1/ST2 and EGFR

WT (gray) and *Areg*$^{-/-}$ (purple) mice were infected with *H. polygyrus*, and on day 14 post infection, mLN were harvested.

(A and B) mLN cells were stimulated with rIL-33, anti-CD3, or media, and the IL-13 (A) and p-ERK (B) expression was determined by intra-cellular staining and flow cytometry analysis.

(C) MHCII-deficient mice were infected with *H. polygyrus* and 7 days post-infection received flow cytometry-sorted CD4$^+$ T cells derived from mLN of naive or *H. polygyrus*-infected WT, *Egfr*$^{fl/fl}$*xCd4-cre*, or *Areg*$^{-/-}$ mice. Worm burden and egg counts were determined 2 weeks post infection.

(D–F) mLN cells were stimulated with rIL-33, rAREG, both, or media only, and EGFR p-Y1068 (D), p-ERK (E), and IL-13 (F) expression was determined by flow cytometry analysis.

(G) EGFR phosphorylation at position Y992 on CD4$^+$ T cells derived from mLN of WT or *Areg*$^{-/-}$ *H. polygyrus*-infected mice in the presence or absence of rAREG.

(H) HEK293T cells were transfected as indicated with T1/ST2 and the IL-1RacP in combination with WT EGFR or EGFR^{Y992F} mutant. Subsequently, the cell lysates were analyzed for the expression of the transfected proteins (input, left panel). The same lysates were also subjected to an EGFR-specific immunoprecipitation (EGFR-IP, right panel) or were treated with the isotype control (iso, right panel). Precipitates were analyzed by immunoblot.

(I) mLN cells were stimulated with rIL-33 in the presence of monensin, and EGFR expression on CD4$^+$ T cells was analyzed by flow cytometry.

All data are representative of at least two independent experiments (mean ± SEM); results for individual mice are shown as dots. See also Figure S7.

express IL-13 at the site of infection in an IL-33-dependent way. This additional AREG-mediated control mechanism may further restrict the interaction of both receptors to Th2 cells.

Finally, our findings further emphasize the importance of signaling hetero-complexes between different cytokine receptor families for the functioning of the immune system. T1/ST2 has already been shown to form a signaling hetero-complex with c-kit, another RTK, on mast cells (Drube et al., 2010). Thus, despite the fact that the molecular mechanism by which T1/ST2 interacts with these RTKs requires further, more detailed

analysis, it appears that signaling hetero-complexes composed of trans-membrane receptors from different signaling families are a more common phenomenon and may add another, so far largely neglected layer of immune regulation. In particular, since the EGFR is expressed on a wide range of leukocytes and since our findings demonstrate that the EGFR can form hetero-complexes with other cytokine receptors to regulate their signal specificity, it is likely that the role of EGFR expression by leukocytes in the functioning of the immune system is underestimated to date. This notion is further supported by the observation that cancer patients treated with EGFR antagonists suffer not only from a wide range of side effects caused by loss of EGFR function on epithelial cells but also become more susceptible to infections (Burtness et al., 2012).

STAR★METHODS

Detailed methods are provided in the online version of this paper and include the following:

- KEY RESOURCES TABLE
- CONTACT FOR REAGENT AND RESOURCE SHARING
- EXPERIMENTAL MODEL AND SUBJECT DETAILS
  - Breeding of experimental animals
- METHOD DETAILS
  - Splenocyte cultures
  - Cell Lines
  - Nematode infections, de-worming and IL-13 delivery
  - *In vivo* delivery of Brefeldin A and duodena digestion
  - Flow Cytometry and FACS-sorting
  - RNA extraction and quantitative real-time PCR
  - T cell enrichment and adoptive transfer experiments
  - Th2 cells generation and RNaseq analysis
  - Immunoprecipitation on T cells
  - Western blots
  - HEK293T transfection and immunoprecipitation
  - Cytokine and *H. polygyrus* antigen ELISA
  - *Ex vivo* re-stimulation conditions
- QUANTIFICATION AND STATISTICAL ANALYSIS
- DATA AND SOFTWARE AVAILABILITY

SUPPLEMENTAL INFORMATION

Supplemental Information includes seven figures and can be found with this article online at https://doi.org/10.1016/j.immuni.2017.09.013.

AUTHOR CONTRIBUTIONS

C.M.M. designed and performed research, analyzed and interpreted data, and wrote the manuscript; S.D., N.B., C.S., and J.C.M. performed experiments; A.J.S. contributed tools, provided expertise, and wrote the manuscript; A.N.M., T.K., M.M., P.J.C., M.S., P.G.F., and R.M.M. contributed tools, provided expertise, and edited the manuscript; D.M.Z. designed the research, analyzed and interpreted data, wrote the manuscript, and funded the study.

ACKNOWLEDGMENTS

We thank Martin Waterfall, Alison Fulton, and Nicola Logan for excellent technical assistance. Work in the D.M.Z. laboratory is supported by the Medical Research Council, grant MR/M011755/1, and the European Union, grant CIG-631413 ("EGF-R for Immunity"). The M.S. laboratory is supported by the Austrian Science Fund (FWF) grants SFB F3518-B20, W1212, and P25925. A.N.M. is funded by the MRC grant U105178805. C.S. is a long-term EMBO Fellow.

Received: August 13, 2016
Revised: March 13, 2017
Accepted: September 21, 2017
Published: October 17, 2017

REFERENCES

Allen, J.E., and Maizels, R.M. (2011). Diversity and dialogue in immunity to helminths. Nat. Rev. Immunol. *11*, 375–388.

Anthony, R.M., Urban, J.F., Jr., Alem, F., Hamed, H.A., Rozo, C.T., Boucher, J.L., Van Rooijen, N., and Gause, W.C. (2006). Memory T(H)2 cells induce alternatively activated macrophages to mediate protection against nematode parasites. Nat. Med. *12*, 955–960.

Anthony, R.M., Rutitzky, L.I., Urban, J.F., Jr., Stadecker, M.J., and Gause, W.C. (2007). Protective immune mechanisms in helminth infection. Nat. Rev. Immunol. *7*, 975–987.

Burtness, B., Marur, S., Bauman, J.E., Golemis, E.A., Mehra, R., and Cohen, S.J. (2012). Comment on "epidermal growth factor receptor is essential for toll-like receptor 3 signaling". Sci. Signal. *5*, lc5.

Camberis, M., Le Gros, G., and Urban, J., Jr. (2003). Animal model of *Nippostrongylus brasiliensis* and *Heligmosomoides polygyrus*. In Current Protocols in Immunology (John Wiley and Sons, Inc.), pp. 19.12.1–19.12.27.

Carvalho, L., Sun, J., Kane, C., Marshall, F., Krawczyk, C., and Pearce, E.J. (2009). Review series on helminths, immune modulation and the hygiene hypothesis: mechanisms underlying helminth modulation of dendritic cell function. Immunology *126*, 28–34.

Cliffe, L.J., Humphreys, N.E., Lane, T.E., Potten, C.S., Booth, C., and Grencis, R.K. (2005). Accelerated intestinal epithelial cell turnover: a new mechanism of parasite expulsion. Science *308*, 1463–1465.

Drube, S., Heink, S., Walter, S., Löhn, T., Grusser, M., Gerbaulet, A., Berod, L., Schons, J., Dudeck, A., Freitag, J., et al. (2010). The receptor tyrosine kinase c-Kit controls IL-33 receptor signaling in mast cells. Blood *115*, 3899–3906.

Endo, Y., Hirahara, K., Iinuma, T., Shinoda, K., Tumes, D.J., Asou, H.K., Matsugae, N., Obata-Ninomiya, K., Yamamoto, H., Motohashi, S., et al. (2015). The interleukin-33-p38 kinase axis confers memory T helper 2 cell pathogenicity in the airway. Immunity *42*, 294–308.

Finney, C.A., Taylor, M.D., Wilson, M.S., and Maizels, R.M. (2007). Expansion and activation of CD4(+)CD25(+) regulatory T cells in Heligmosomoides polygyrus infection. Eur. J. Immunol. *37*, 1874–1886.

Grencis, R.K. (2015). Immunity to helminths: resistance, regulation, and susceptibility to gastrointestinal nematodes. Annu. Rev. Immunol. *33*, 201–225.

Guo, L., Wei, G., Zhu, J., Liao, W., Leonard, W.J., Zhao, K., and Paul, W. (2009). IL-1 family members and STAT activators induce cytokine production by Th2, Th17, and Th1 cells. Proc. Natl. Acad. Sci. USA *106*, 13463–13468.

Guo, L., Huang, Y., Chen, X., Hu-Li, J., Urban, J.F., Jr., and Paul, W.E. (2015). Innate immunological function of TH2 cells in vivo. Nat. Immunol. *16*, 1051–1059.

Hasnain, S.Z., Evans, C.M., Roy, M., Gallagher, A.L., Kindrachuk, K.N., Barron, L., Dickey, B.F., Wilson, M.S., Wynn, T.A., Grencis, R.K., and Thornton, D.J. (2011). Muc5ac: a critical component mediating the rejection of enteric nematodes. J. Exp. Med. *208*, 893–900.

Hewitson, J.P., Filbey, K.J., Grainger, J.R., Dowle, A.A., Pearson, M., Murray, J., Harcus, Y., and Maizels, R.M. (2011). Heligmosomoides polygyrus elicits a dominant nonprotective antibody response directed against restricted glycan and peptide epitopes. J. Immunol. *187*, 4764–4777.

Johnston, C.J., Robertson, E., Harcus, Y., Grainger, J.R., Coakley, G., Smyth, D.J., McSorley, H.J., and Maizels, R. (2015). Cultivation of Heligmosomoides polygyrus: an immunomodulatory nematode parasite and its secreted products. J. Vis. Exp. *98*, e52412.

Liao, W., Schones, D.E., Oh, J., Cui, Y., Cui, K., Roh, T.Y., Zhao, K., and Leonard, W.J. (2008). Priming for T helper type 2 differentiation by interleukin

2-mediated induction of interleukin 4 receptor alpha-chain expression. Nat. Immunol. *9*, 1288–1296.

Liu, F., and Whitton, J.L. (2005). Cutting edge: re-evaluating the in vivo cytokine responses of CD8+ T cells during primary and secondary viral infections. J. Immunol. *174*, 5936–5940.

Löhning, M., Stroehmann, A., Coyle, A.J., Grogan, J.L., Lin, S., Gutierrez-Ramos, J.C., Levinson, D., Radbruch, A., and Kamradt, T. (1998). T1/ST2 is preferentially expressed on murine Th2 cells, independent of interleukin 4, interleukin 5, and interleukin 10, and important for Th2 effector function. Proc. Natl. Acad. Sci. USA *95*, 6930–6935.

McKenzie, G.J., Bancroft, A., Grencis, R.K., and McKenzie, A.N. (1998). A distinct role for interleukin-13 in Th2-cell-mediated immune responses. Curr. Biol. *8*, 339–342.

Meulenbroeks, C., van Weelden, H., Schwartz, C., Voehringer, D., Redegeld, F.A.M., Rutten, V.P.M.G., Willemse, T., Sijts, A.J.A.M., and Zaiss, D.M.W. (2015). Basophil-derived amphiregulin is essential for UVB irradiation-induced immune suppression. J. Invest. Dermatol. *135*, 222–228.

Minutti, C.M., Jackson-Jones, L.H., García-Fojeda, B., Knipper, J.A., Sutherland, T.E., Logan, N., Rinqvist, E., Guillamat-Prats, R., Ferenbach, D.A., Artigas, A., et al. (2017). Local amplifiers of IL-4Rα-mediated macrophage activation promote repair in lung and liver. Science *356*, 1076–1080.

Monticelli, L.A., Osborne, L.C., Noti, M., Tran, S.V., Zaiss, D.M., and Artis, D. (2015). IL-33 promotes an innate immune pathway of intestinal tissue protection dependent on amphiregulin-EGFR interactions. Proc. Natl. Acad. Sci. USA *112*, 10762–10767.

Moro, K., Yamada, T., Tanabe, M., Takeuchi, T., Ikawa, T., Kawamoto, H., Furusawa, J., Ohtani, M., Fujii, H., and Koyasu, S. (2010). Innate production of T(H)2 cytokines by adipose tissue-associated c-Kit(+)Sca-1(+) lymphoid cells. Nature *463*, 540–544.

Neill, D.R., Wong, S.H., Bellosi, A., Flynn, R.J., Daly, M., Langford, T.K., Bucks, C., Kane, C.M., Fallon, P.G., Pannell, R., et al. (2010). Nuocytes represent a new innate effector leukocyte that mediates type-2 immunity. Nature *464*, 1367–1370.

Nosbaum, A., Prevel, N., Truong, H.A., Mehta, P., Ettinger, M., Scharschmidt, T.C., Ali, N.H., Pauli, M.L., Abbas, A.K., and Rosenblum, M.D. (2016). Cutting edge: regulatory T cells facilitate cutaneous wound healing. J. Immunol. *196*, 2010–2014.

Okoye, I.S., Coomes, S.M., Pelly, V.S., Czieso, S., Papayannopoulos, V., Tolmachova, T., Seabra, M.C., and Wilson, M.S. (2014). MicroRNA-containing T-regulatory-cell-derived exosomes suppress pathogenic T helper 1 cells. Immunity *41*, 89–103.

Oliphant, C.J., Hwang, Y.Y., Walker, J.A., Salimi, M., Wong, S.H., Brewer, J.M., Englezakis, A., Barlow, J.L., Hams, E., Scanlon, S.T., et al. (2014). MHCII-mediated dialog between group 2 innate lymphoid cells and CD4(+) T cells potentiates type 2 immunity and promotes parasitic helminth expulsion. Immunity *41*, 283–295.

Pahl, A., Zhang, M., Kuss, H., Szelenyi, I., and Brune, K. (2002). Regulation of IL-13 synthesis in human lymphocytes: implications for asthma therapy. Br. J. Pharmacol. *135*, 1915–1926.

Reynolds, L.A., Filbey, K.J., and Maizels, R.M. (2012). Immunity to the model intestinal helminth parasite Heligmosomoides polygyrus. Semin. Immunopathol. *34*, 829–846.

Saenz, S.A., Siracusa, M.C., Perrigoue, J.G., Spencer, S.P., Urban, J.F., Jr., Tocker, J.E., Budelsky, A.L., Kleinschek, M.A., Kastelein, R.A., Kambayashi, T., et al. (2010). IL25 elicits a multipotent progenitor cell population that promotes T(H)2 cytokine responses. Nature *464*, 1362–1366.

Slifka, M.K., and Whitton, J.L. (2000). Antigen-specific regulation of T cell-mediated cytokine production. Immunity *12*, 451–457.

Smith, K.A., Filbey, K.J., Reynolds, L.A., Hewitson, J.P., Harcus, Y., Boon, L., Sparwasser, T., Hämmerling, G., and Maizels, R.M. (2016). Low-level regulatory T-cell activity is essential for functional type-2 effector immunity to expel gastrointestinal helminths. Mucosal Immunol. *9*, 428–443.

Taylor, B.C., Zaph, C., Troy, A.E., Du, Y., Guild, K.J., Comeau, M.R., and Artis, D. (2009). TSLP regulates intestinal immunity and inflammation in mouse models of helminth infection and colitis. J. Exp. Med. *206*, 655–667.

Urban, J.F., Jr., Madden, K.B., Svetić, A., Cheever, A., Trotta, P.P., Gause, W.C., Katona, I.M., and Finkelman, F.D. (1992). The importance of Th2 cytokines in protective immunity to nematodes. Immunol. Rev. *127*, 205–220.

Wynn, T.A., and Ramalingam, T.R. (2012). Mechanisms of fibrosis: therapeutic translation for fibrotic disease. Nat. Med. *18*, 1028–1040.

Zaiss, D.M., Yang, L., Shah, P.R., Kobie, J.J., Urban, J.F., and Mosmann, T.R. (2006). Amphiregulin, a TH2 cytokine enhancing resistance to nematodes. Science *314*, 1746.

Zaiss, D.M., van Loosdregt, J., Gorlani, A., Bekker, C.P., Gröne, A., Sibilia, M., van Bergen en Henegouwen, P.M., Roovers, R.C., Coffer, P.J., and Sijts, A.J. (2013). Amphiregulin enhances regulatory T cell-suppressive function via the epidermal growth factor receptor. Immunity *38*, 275–284.

Zaiss, D.M.W., Gause, W.C., Osborne, L.C., and Artis, D. (2015). Emerging functions of amphiregulin in orchestrating immunity, inflammation, and tissue repair. Immunity *42*, 216–226.

STAR★METHODS

KEY RESOURCES TABLE

REAGENT or RESOURCE	SOURCE	IDENTIFIER
Antibodies		
Rat anti mouse CD4 (clone RM4-5)	Biolegend	Cat#100536
Armenian Hamster anti mouse CD69 (clone H1.2F3)	Biologend	Cat#104528
Anti mouse T1/ST2 (clone DJ8)	MD Bioproducts	Cat#101001B
Rat anti mouse CD62L (clone MEL-14)	Biolegend	Cat#104424
Rat anti CD44 (clone IM7)	Biolegend	Cat#103032
Rat anti mouse CD45Rb (clone 16A)	BD PharMingen	Cat#553101
Rat anti mouse CD25 (clone PC61)	Biolegend	Cat#102016
Mouse anti mouse CD45.1 (clone A20)	eBiosciences	Cat#17-0453-81
Mouse anti mouse CD45.2 (clone 104)	eBiosciences	Cat#12-0454-81
Rat anti mouse CD8 (clone 53-6.7)	Biolegend	Cat#100706
Rat anti mouse CD19 (clone 6D5)	Biolegend	Cat#115538
Rat anti mouse SiglecF (clone E50-2440)	BD PharMingen	Cat#562681
Rat anti mouse Ly6G (clone 1A8)	Biolegend	Cat#127628
Rat anti mouse CD3 (clone 17A2)	Biolegend	Cat#100228
Mouse anti mouse CD64 (clone X54-5/7.1)	Biolegend	Cat#139306
Rat anti mouse Ly6C (clone HK1.4)	Biolegend	Cat#128024
Armenian Hamster anti mouse CD11c (clone N418)	Biolegend	Cat#117334
Rat anti CD11b (clone M1/70)	Biolegend	Cat#101241
Rat anti mouse F4/80 (clone BM8)	eBiosciences	Cat#25-4801-82
Rat anti mouse MHCII (I-A/I-E) (clone M5/114.15.2)	eBiosciences	Cat#47-5321-82
Rat anti mouse CD90.2 (clone 30-H12)	Biolegend	Cat#105308
Armenian Hamster anti mouse FcεR1 (clone MAR-1)	Biolegend	Cat#134309
Mouse anti mouse NK-1.1 (clone PK136)	Biolegend	Cat#108710
Mouse anti EGF-R (clone EGF-R1)	Abcam	Cat#Ab30
Rat anti mouse IL-13 (clone eBio13A)	eBiosciences	Cat#50-7133-80
Rat anti mouse IL-4 (clone 11B11)	Biolegend	Cat#504104
Rat anti IL-5 (clone TRFK5)	BD PharMingen	Cat#554395
Rat anti mouse IFN-γ (clone XMG1.2)	Biolegend	Cat#505806
Rabbit polyclonal anti p-p44/42 MAPK (Erk1/2) (Thr202/Tyr204)	Cell Signaling	Cat#9101
Mouse anti IκBα (clone L35A5)	Cell Signaling	Cat#4814
Rabbit polyclonal anti p-EGFR (Tyr1068)	Cell Signaling	Cat# (D7A5) XP 3777
Rabbit polyclonal anti p-EGFR (Tyr992)	ThermoFisher	Cat#44-786G
Rat anti FoxP3 (clone FJK-16 s)	eBiosciences	Cat#50-5773-82
Human anti GATA3 (clone REA174)	Miltenyi Biotec	Cat#130-108-061
Mouse anti c-Maf (clone sym0F1)	eBioscience	Cat#12-9855-41
Rat anti mouse CD8 (clone 53-6.7)	BioXcell	Cat#BE0004-1
Rat anti mouse B220 (clone RA3.3A1/6.1 (TIB-146))	BioXcell	Cat#BE0067
Rat anti mouse CD11b (clone M1/70)	BioXcell	Cat#BE0007
Rat anti mouse MHCII (I-A/I-E) (clone M5/114)	BioXcell	Cat#BE0108
Armenian Hamster anti mouse CD3 (clone 145-2C11)	BD PharMingen	Cat#553058
Rabbit polyclonal anti T1/ST2	Thermo Scientific/Pierce	Cat#PA5-20077
Rabbit polyclonal anti STAT6	Cell Signaling	Cat#9362
Anti EGFR (clone EP38Y)	Abcam	Cat#ab52894
Rabbit polyclonal hEGFR	Santa Cruz Biotechnology	Cat#sc-03-G

(*Continued on next page*)

Continued

REAGENT or RESOURCE	SOURCE	IDENTIFIER
Rabbit polyclonal anti Sos	Cell Signaling	Cat#5890
Rabbit polyclonal anti SHP-2	Cell Signaling	Cat#3752
Goat polyclonal anti hIL-33R	R&D systems	Cat#Baf523
Anti-hIL-1RacP	R&D systems	Cat#AF676
Bacterial and Virus Strains		
Heligmosomoides polygyrus	Camberis et al., 2003	N/A
Mouse-adapted *Nippostrongylus brasiliensis*	Minutti et al., 2017	N/A
Chemicals, Peptides, and Recombinant Proteins		
Brefeldin A	Sigma	Cat#B6542
Recombinant mouse IL-13	Immunotools	Cat#12340137
Recombinant mouse IL-33	PeproTech	Cat#210-33
Recombinant human HB-EGF	R&D Systems	Cat#259-HE
Recombinant mouse IL-7	PeproTech	Cat#217-17
Recombinant mouse IL-2	BD Biosciences	Cat#550069
Recombinant mouse TSLP	eBiosciences	Cat#34-8498-82
MEK inhibitor	Promega	Cat#V1121
Gefitinib	LC laboratories	Cat#G-4408
Marimastat	Abcam	Cat#Ab141276
OVA	Sigma-Aldrich	Cat#A5503
Critical Commercial Assays		
LIVE/DEAD Fixable Blue Dead Cell Stain Kit	Thermo Fisher	Cat#L23105
Sheep anti-Rat IgG Dynabeads	Invitrogen	Cat#110.35
IL-13 Mouse ELISA Kit	eBioscience	Cat#88-7137-76
IL-5 Mouse ELISA Kit	eBioscience	Cat#88-7054-88
Deposited Data		
Sequencing raw data of RNA-seq experiment	GEO accession number	GEO: GSE104096
Experimental Models: Cell Lines		
HEK293 cells	ATCC	N/A
HaCaT cells	ATCC	N/A
Experimental Models: Organisms/Strains		
EGFR$^{flox/flox}$	Natarajan A et al., 2007 PNAS 104:17081	N/A
CD4-Cre	Taconics	Stock No: 4196
FoxP3-Cre	The Jackson Laboratory	Stock No: 016959
Areg$^{-/-}$	Luetteke NC et al., 1999 Development. 126:2739-50	N/A
MHCII$^{-/-}$ (B6;129S2-H2dlAb1-Ea/J)	The Jackson Laboratory	Stock No: 003374
T1/ST2$^{-/-}$	Townsend MJ et al., 2000 J Exp Med. 191:1069	N/A
IL-13$^{-/-}$	McKenzie et al., 1998	N/A
Oligonucleotides		
Primer for *Il13* mRNA	Applied Biosystems	Cat#Mm00434204
Primer for *Il5* mRNA	Applied Biosystems	Cat#Mm00439646
Primer for *Il4* mRNA	Applied Biosystems	Cat#Mm00445259
Primer for *Cd3e* mRNA	Applied Biosystems	Cat#Mm00599684
Primer for *Muc5ac* mRNA	Applied Biosystems	Cat#Mm01276718
Primer for *Areg* mRNA	Applied Biosystems	Cat#Mm00437583
Primer for *Rn18s* mRNA	Applied Biosystems	Cat#Mm03928990

(*Continued on next page*)

Continued		
REAGENT or RESOURCE	SOURCE	IDENTIFIER
Recombinant DNA		
pcDNA3-hIL-33R		N/A
pcDNA3-hIL-1RacP		N/A
pRK5-wt-hEGFR		N/A
pRK5-hY992F-EGFR		N/A
Software and Algorithms		
FlowJo 10	FLOWJO, LLC	https://www.flowjo.com/
Prism 7	GraphPad Software	https://www.graphpad.com/scientific-software/prism/

CONTACT FOR REAGENT AND RESOURCE SHARING

Further information and request for resources and reagents should be directed to and will be fulfilled by the Lead Contact, Dietmar Zaiss (dietmar.zaiss@ed.ac.uk).

EXPERIMENTAL MODEL AND SUBJECT DETAILS

Breeding of experimental animals

C57BL/6J mice (*wt*, $Egfr^{flox/flox}$, $Cd4$-cre x $Egfr^{flox/flox}$, *Foxp3*-cre x $Egfr^{flox/flox}$, $Areg^{-/-}$ and MHCII-deficient $A\beta^{-/-}$) were bred and maintained at the University of Edinburgh, Trinity College Dublin (T1/ST2-deficient $Il1rl1^{-/-}$) or the Laboratory of Molecular Biology, Cambridge ($Il13^{-/-}$) in specific-pathogen free conditions. Sex-matched mice were 6-8-weeks old at the start of the experiment, and all mice were housed in individually ventilated cages. Mice were not randomized in cages, but each cage was randomly assigned to a treatment group. Investigators were not blinded to mouse identity during necropsy but during worm and egg counts. Experiments were performed in accordance with the United Kingdom Animals (Scientific Procedures) Act of 1986. All researchers were accredited by the UK government Home Office. Dispensation to carry out animal research at The University of Edinburgh was approved by the University of Edinburgh Animal Welfare and Ethical Review Body and granted by the UK government Home Office; as such all research was carried under the project licenses PPL70/8470 and 70/8483. All animal experiments in Ireland were performed under license in compliance with the Health Products Regulatory Authority and approved by the Trinity College Dublin's BioResources ethical review board. Note: Male and female mice were used to perform the experiments shown in this manuscript. However, none of the experiments were conducted using both sexes at the same time. We never observed an obvious difference between sexes within the parameters analyzed for our experiments.

METHOD DETAILS

Splenocyte cultures

Single cell suspensions of spleens were obtained by forcing the tissue through a 70 μM cell strainer. Subsequently, cells were treated with red blood cell lysis buffer (Sigma-Aldrich) and counted using an automated cellometer T4 (Peqlab, Radnor, PA). In some experiments, T helper cells were sorted based on CD3 and CD4 positivity and different T cell subpopulations were sorted as indicated. Cells were incubated in IMDM medium supplemented with 10% FCS, 1% l-glutamine, 1% penicillin/streptomycin and 5×10^{-5} M 2-mercaptoethanol at 37°C in a humidified atmosphere at 5% CO_2.

Cell Lines

HEK293 cells and HaCaT cells were cultured in IMDM medium supplemented with 10% FCS, 1% l-glutamine, 1% penicillin/streptomycin and 5×10^{-5} M 2-mercaptoethanol at 37°C in a humidified atmosphere at 5% CO_2.

Nematode infections, de-worming and IL-13 delivery

H. polygyrus and mouse-adapted *N. brasiliensis* were maintained by serial passage trough F1 (C57BL/6xCBA) and C57BL/6 mice respectively, as described previously (Camberis et al., 2003; Minutti et al., 2017). Mice were infected by oral gavage with 200 *H. polygyrus* and subcutaneously with 250 *N. brasiliensis* third-stage larvae. For deworming, mice were treated orally with pyrantel embonate in the form of 2.5 mg Strongid P paste (Pfizer) in 0.2 mL water on day 14 post-primary infection (Hewitson et al., 2011). In IL-13 delivery experiments, 5 μg of rmIL-13 (Immunotools) was injected intra-peritoneally at days 6, 7 and 8 post *H. polygyrus* infection. Egg output was analyzed in faeces and adult worm burdens were determined by removing the small intestine and exposing the lumen by dissection.

In vivo delivery of Brefeldin A and duodena digestion

In vivo delivery of Brefeldin A was performed as previously described (Liu and Whitton, 2005). Briefly, Brefeldin A (Sigma) was resuspended at 20 mg/ml in DMSO. Further dilution to 1 mg/ml was made in PBS, and 250 μL were injected i.v.. 6 hours later duodena were harvested and intestial-resident leukocytes were purified by two sequantial pre-digestions (20 mins, at 37C) with HBSS containing: 10 mM HEPES, 5 mM EDTA, 1 mM DTT and 5% FCS in the presence or absence of Monensin (Invitrogen) (10 μM). After, samples were digested for 30 mins at 37C with HBSS containing 10 mM HEPES, 0.5 mg/mL Collagenase D, 0.5 mg/mL DNase I grade II, 3 mg/mL Dispase II (Roche) and 5% FCS. Single cell suspensions were prepared by forcing the samples through a 70-μm cell stainer and subsequent washes.

Flow Cytometry and FACS-sorting

Cells were incubated with Fc block (CD16/CD32 and 10% mouse serum) and stained with a combination of the following commercial monoclonal fluorescently conjugated antibodies (clone, brand and catalog no. specified in the KEY RESOURCES TABLE): CD4, CD69, T1/ST2, CD62L, CD44, CD45Rb, CD25, CD45.1, CD45.2, CD8, CD19, SiglecF, Ly6G, CD3, CD64, Ly6C, CD11c, CD11b, F4/80, I-A/I-E (MHCII), CD90.2, FcεR1, NK-1.1 and EGF-R. For intracellular staining, cells were fixed with 2% paraformaldehyde in dPBS for 20 min at room temperature, permeabilized with 0.5% Saponin or ice-cold methanol then stained with IL-13, IL-4, IL-5, IFN-γ, p-p44/42 MAPK (Erk1/2) (Thr202/Tyr204), IκBα, p-EGFR (Tyr1068), p-EGFR (Tyr992) or isotype control followed by anti-Rabbit IgG secondary antibodies (Invitrogen). For detection of FoxP3, GATA3 and c-Maf cells were stained for surface markers then fixed and permeabilized using FoxP3 staining buffer set (eBioscience). Cells were then stained with FoxP3, GATA3 and c-Maf for 30 min at room temperature. Live/Dead (Life Technologies) was used to exclude dead cells from analysis. Samples were analyzed by flow cytometry using Becton Dickinson FACS LSR II and FlowJo software. Alveolar macrophages were identified as described before (Minutti et al., 2017): lineage negative^{-} (CD19^{-}, Ly6G^{-} and CD3^{-}), CD64^{+}, CD11c^{+} and SiglecF^{+}. ILC2 cells were identified as described before (Monticelli et al., 2015), detailed gating strategy is shown in Figure S4E.

For some experiments, T cells from mLN of infected mice were enriched (as described below) and subsequently stained with a combination of antibodies to CD3, CD4, CD69, T1/ST2, CD62L, CD44, CD45Rb, CD25 and EGF-R prior to sorting on a FACS Aria (BD). T helper cells were sorted based on CD3 and CD4 positivity and different T cell subpopulations were sorted as indicated. T cell purity was verified by flow cytometry.

RNA extraction and quantitative real-time PCR

Tissue was homogenized in TRIzol with a TissueLyser (QIAGEN) and RNA was isolated following manufacturer's instructions. Reverse transcription was performed using 1 μg of total RNA using 200 U of M-MLV reverse transcriptase, 10 mM dNTPs, and 0.5 μg Oligo dT15 and RNasin inhibitor (Promega). Expression of genes of interest was measured by real-time PCR with the Lightcycler 480 II system (Roche) using Taqman Master kit and specific primers (specified in the KEY RESOURCES TABLE), as previously described (Meulenbroeks et al., 2015). PCR amplification was analyzed using 2nd derivative maximum algorithm (LightCycler 480 Sw 1.5, Roche) and the expression of the gene of interest was normalized to the housekeeping gene *Rn18s*.

T cell enrichment and adoptive transfer experiments

For transfer experiments, mLN were collected from donor mice 14 days after *H. polygyrus* infection. mLN single cell suspensions were prepared by forcing through a 70uM cell strainer and the homogenates were treated to lysate red blood cells. For transfer of CD4^{+} T cells, these cells were purified by negative selection using Sheep anti-Rat IgG Dynabeads (Invitrogen) and monoclonal antibodies (specified in the KEY RESOURCES TABLE) to CD8, B220, CD11b and I-A/I-E (MHCII) following manufacturer's protocol. Approximately 1.5 x10^{6} Cells were transferred intravenously. In some experiments, CD4^{+} T cell subpopulations were further purified by FACS-sorting prior to intra-venous transfer, in this case approximately 1 x10^{5} cells were transferred. All recipients received the same percentage of Th2 cells as determined by the analysis of GATA3 expression and/or IL-13 expression by re-stimulated CD4^{+} T cells. *H. polygyrus*-infected recipient mice received transferred cells on day 7 post-infection whereas *N. brasiliensis*-infected recipients mice received transferred cells at the same time of infection.

For the generation of resting T cells, donor mice were infected with *H. polygyrus* and two weeks after infection the mice were dewormed by oral deliver of pyrantel embonate in the form of 2.5 mg Strongid P paste in 0.2 mL water. After two weeks of deworming treatment mLNs were harvested and T cells were enriched as described above. Ova-specific activated and memory T cells were generated with nebulised ovalbumin. In brief, animals were immunized twice by an intra-peritoneal injection of 10ug of OVA (Sigma-Aldrich) dissolved in alum, with a break of two weeks between each immunization. Seven days following the second immunisation, mice were nebulised for 20 minutes on 5 consecutive days, with either PBS only, or with PBS containing 100ug/ml of ova, at a rate of 1 ml/minute. Thoracic lymph nodes (parathymic, posterior, mediastinal and paravertebral LN) were collected 24 h or 1 month following the last treatment to isolate activated or memory CD4^{+} T cells, respectively. T cells were FACS sorted and transferred as indicated with or without concomitant ip. delivery of ova/alum or adjuvant only.

Th2 cells generation and RNaseq analysis

CD4^{+} CD25^{-} CD45Rb^{hi} cells were FACS-sorted from spleens of *wt* and EGFR$^{\Delta CD4}$ mice and cultured in IMDM medium supplemented with 10% FCS, 1% l-glutamine, 1% penicillin/streptomycin and 5×10^{-5} M 2-mercaptoethanol. Th2 cells were generated

by adding IL-2 (1 ng/mL), IL-4 (2 ng/ml) and neutralizing IFN-γ antibody (clone R4.6A2, 5 μg/mL) into the culture. RNA was isolated using the RNeasy Mini Kit (QUIAGEN Cat. No. 74104). RNA seq data are deposited under GEO accession number: GSE104096.

Immunoprecipitation on T cells

$CD4^+$ T cells were enriched by negative selection using Sheep anti-Rat IgG Dynabeads as described above. Purified T cells were stimulated with IL-33 (PeproTech) (10 ng/mL) or vehicle for 1 hour. After culture, cells were lysed at 4°C for 30 min in 500 μL of lysis buffer containing: 50 mM Tris-HCl (pH 7.6), 150 mM NaCl, 5 mM EDTA, 1% NP-40, 0.05% Sodium azide and 1 mM phenylmethylsulfonyl fluoride (PMSF). The lysates were centrifuged at 10 000 × g for 10 min, and the supernatants were pre-cleared by adding protein A-Sepharose (50 μL) and incubated at 4°C for 1 hour, followed by centrifugation at 10 000 × g for 10 min. The pre-cleared supernatant was incubated with anti-T1/ST2 antibody (Thermo Scientific/Pierce, 10ug/ml) or control IgG at 4°C overnight, after which 50 μL of protein A-Sepharose was added for 4 h at 4°C with gentle rotation. The immune complexes were collected by centrifugation at 10 000 × g for 5 min at 4°C, washed three times with cold lysis buffer, and released by boiling with 5 × Laemmli loading buffer.

Western blots

Cell lysates and samples from co-immunoprecipitation assays were resolved by 8% (m/v) SDS-PAGE in reducing conditions and transferred to nitrocellulose membranes. After blocking with 2% BSA, membranes were washed and incubated with an anti-Stat6, EGFR or T1/ST2 antibodies (brand and catalog no. specified in the KEY RESOURCES TABLE) overnight at 4°C. The membranes were washed and incubated with horseradish-peroxidase-labeled secondary antibodies for 1 hour at room temperature and exposed to ECL reagents.

HEK293T transfection and immunoprecipitation

HEK293T cells (0.8 x10^6 cells/sample) were seeded in DEMEM (Sigma) supplemented with 10% FCS but w/o antibiotics. Cells were incubated overnight (according to the lipofectamin transfection protocol). Cells were transfected with pcDNA3-hIL-33R, pcDNA3-hIL-1RacP, pRK5-wt-hEGFR or pRK5-hY992F-EGFR (1.3μg DNA/sample of each construct) (pRK5-wt-hEGFR and pRK5-hY992F-EGFR were kindly provided by Prof. Dr. F.-D. Böhmer; Department for Moleculare Cell biology, Jena) by using lipofectamin (Invitrogen) according to the manufacturer procedures. To obtain the same DNA content in every sample (total 4μg DNA/sample), single- and co-transfections were filled with empty vector (pcDNA3). Subsequently, HEK293T cells were incubated for 24h. Afterward, cells were lysed (with Lysis buffer containing: 20 mM HEPES, pH7.5; 10 mM EDTA; 40 mM β-glycerophosphate; 2,5 mM MgCl2; 2 mM orthovanadate; 1 mM dithiothreitol; 20 μg/ml aprotinin; 20 μg/ml leupeptin supplemented with 0,5% NP40). Protein concentration was determined by using the BCA-kit (Pierce). A small aliquot of every sample was taken to perform the input blot. Thereby, samples were treated and boiled in 6 x Laemmli buffer. Samples were analyzed by SDS-PAGE (8%) and western blotting by using the anti-hEGFR, the anti-Sos, the anti-Shp2, the anti-hIL-33R and the anti-hIL-1RacP antibodies (brand and catalog no. specified in the KEY RESOURCES TABLE). The rest of the samples were subjected to anti-hEGFR-specific or to an isotype (as non-specific goat control immunoglobulins; Gentaur) immunprecipitation overnight. Samples were treated with Protein-G sepharoses (invtrogen) for 4-6h. Precipitates were washed with Lysisbuffer and PBS, and were treated and boiled in 6 x Laemmli buffer. Immunoprecipitated samples were analyzed by SDS-PAGE (8%) and western blotting by using anti-hEGFR, anti-Sos, anti-Shp2, anti-hIL-33R and anti-hIL-1RacP antibodies. As secondary antibodies for all western blots we used anti-rabbit-POD (for the detection of hEGFR, Sos, Shp2 and hIL-1Racp) (Santa Cruz Biotechnology). For detection of the hIL-33R we a used anti-goat-POD (Santa Cruz Biotechnology) or POD-coupled streptavidine (Roche). Membranes were developed with the ECL-reagent (Pierce).

Cytokine and *H. polygyrus* antigen ELISA

Mouse IL-13 and IL-5 were measured according to manufacturers instructions using ELISA Kits (eBioscience). *H. polygyrus* antigen (HEX; Finney et al., 2007) was prepared by homogenizing adult worms in PBS, which was centrifuged (13 000 × g, 10 min); the supernatant was filtered (0.2 μm) and stored at 1.5 mg/mL at –80°C. Antigen-specific antibody responses were determined by ELISA as described before (Finney et al., 2007). Multisorp (Nunc) plates were coated with 5 μg/mL *H. polygyrus* antigen in 0.06 M carbonate buffer pH 9.6, overnight at 4°C. Plates were blocked with 5% BSA for 2 h at 37°C. Faecal homogenates and sera were diluted in TBS/0.05% Tween and added to wells overnight at 4°C. Antigen-specific Ig isotypes were detected with HRP-conjugated detecting antibodies (Southern Biotechnology), with TMB peroxidase substrate.

Ex vivo re-stimulation conditions

To determine the induction of cytokines expression, FACS-sorted T cells and mLN cells or splenocytes were cultured in 96-well plates in IMDM medium, 10% FCS, 1% l-glutamine, 1% penicillin/streptomycin (GIBCO), 5×10^{-5} M 2-mercaptoethanol in the presence or absence of Monoensin (Invitrogen) (10 μM) and rmIL-33 (PeproTech) (10 ng/mL) or anti-CD3 (BD, 145-2C11) (2 μg/mL) for six hours. Alternatively, intracellular amounts of p-EGFR (Y1068 and Y992), p-ERK and IκBα were analyzed after 30 minutes of stimulation with IL-33 (10 ng/mL), anti-CD3 (2 μg/mL), AREG (R&D) (10 ng/mL) or HB-EGF (R&D) (10 ng/mL). To analyze EGFR or AREG induction, splenocytes or FACS-sorted T cells were re-stimulated overnight with *H. polygyrus* excretory-secretory products (HES; Johnston et al., 2015) (1 μg/mL), adult worm extract (HEX) (10 μg/mL), anti-CD3 (2 μg/mL), IL-7 (PeproTech) (20 ng/mL), IL-2 (BD) (1 ng/mL), TSLP (eBiosciences) (20 ng/mL) and IL-33 (10 ng/mL). Inhibitors were used at the following concentrations: MEK inhibitor (Promega)(10 μM), Gefitinib (LC laboratories) (1 μM) and Marimastat (AbCam) (25 μM). The inhibitory effect of

Marimastat (25 μM) was checked by its ability to prevent the release of activated Amphiregulin by transfected HEK cells. Under these conditions cell viability was higher than 90% as compared to 97% at the start of the experiment.

QUANTIFICATION AND STATISTICAL ANALYSIS

Normal distribution of data was determined by visual examination of residuals. Statistical evaluation of different groups was performed either by analysis of variance (ANOVA) followed by the Turkey multiple comparison test or by non-parametric Mann-Whitney test, as indicated. An α threshold $\leq$ 5% ($p \leq 0.05$) was considered significant. All statistical calculations were performed using PRISM, (Graphpad, La Jolla, CA).

DATA AND SOFTWARE AVAILABILITY

The accession number for the RNA-seq dataset reported in this paper is GEO: GSE104096.

Immunity, Volume *47*

Supplemental Information

Epidermal Growth Factor Receptor Expression Licenses Type-2 Helper T Cells to Function in a T Cell Receptor-Independent Fashion

Carlos M. Minutti, Sebastian Drube, Natalie Blair, Christian Schwartz, Jame C. McCrae, Andrew N. McKenzie, Thomas Kamradt, Michal Mokry, Paul J. Coffer, Maria Sibilia, Alice J. Sijts, Padraic G. Fallon, Rick M. Maizels, and Dietmar M. Zaiss

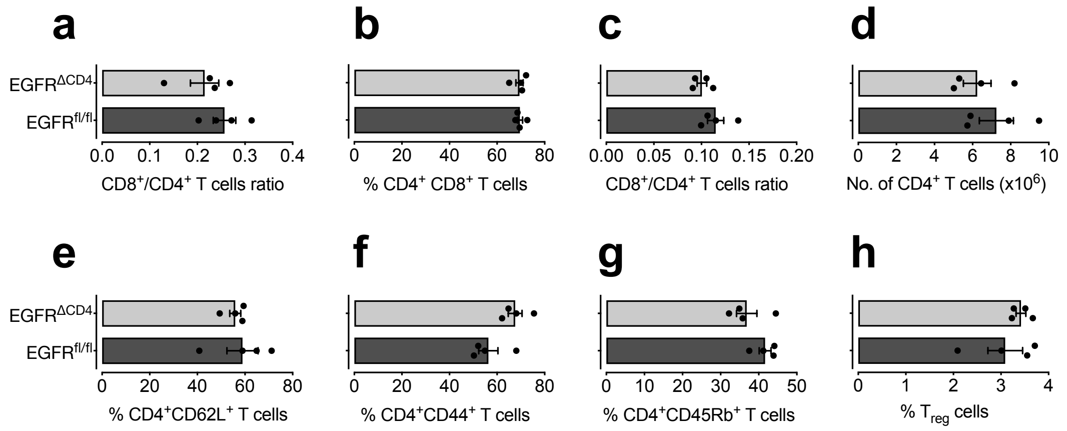

Supplementary Figure S1. Related to Figure 1.

Characterization of naive EGFR$^{\Delta CD4}$ mice. Thymi and spleens were harvested from naïve *wt* and EGFR$^{\Delta CD4}$ mice. **a)** Ratio of CD4 and CD8 single positive T-cells and **b)** frequency of CD4, CD8 double positive T-cells from thymus was determined by FACS. **c)** Ratio of CD4^{+} and CD8^{+} positive T-cells and **d)** frequency of CD4^{+} T-cells from spleens as determined by FACS. Characterization of different CD4^{+} T-cell sub-populations from spleens: **e)** CD62L^{+}, **f)** CD44^{+}, **g)** CD45Rb^{+} and **h)** CD25^{+} FoxP3^{+} regulatory T-cells. Data are representative of two independent experiments (mean ± SEM); results for individual mice are shown as dots.

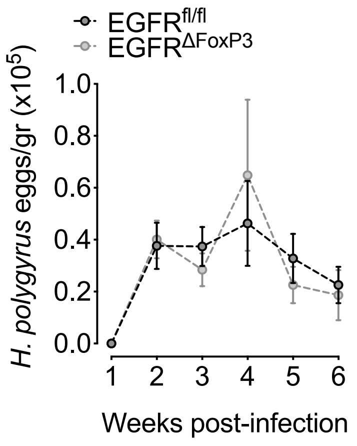

Supplementary Figure S2. Related to Figure 1.

EGFR expression by regulatory T cells does not contribute to worm clearance. WT and EGFR$^{\Delta FoxP3}$ mice were infected with *H. polygyrus* larvae or left untreated. Egg count in faeces were analysed at different times following infection with *H. polygyrus* (n = 3 mice). Data are representative of two independent experiments (mean ± SEM).

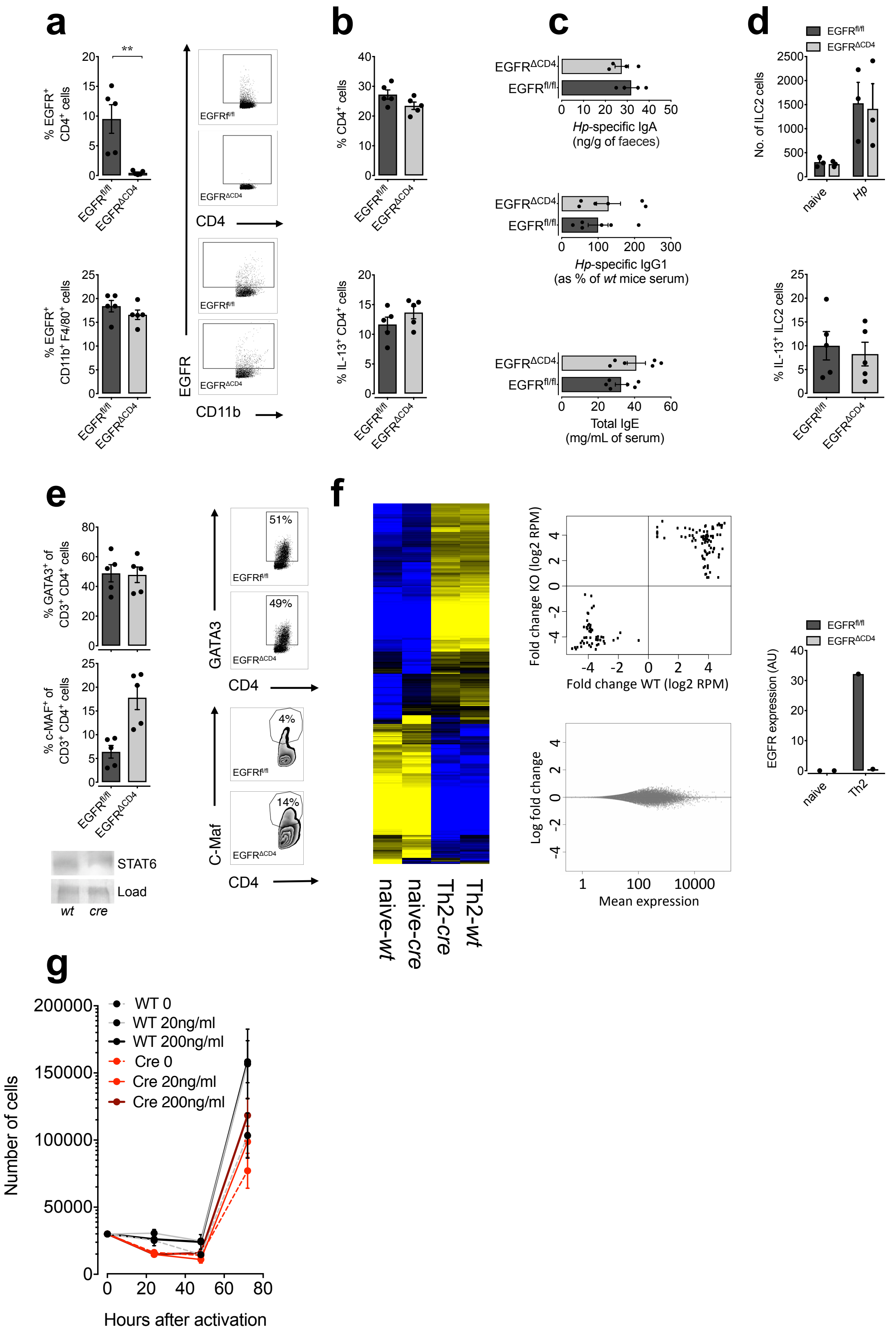
a
% EGFR+ CD4+ cells
**
EGFRfl/fl
EGFRΔCD4
% EGFR+ CD11b+ F4/80+ cells
EGFR
CD4
CD11b
b
% CD4+ cells
% IL-13+ CD4+ cells
c
Hp-specific IgA (ng/g of faeces)
Hp-specific IgG1 (as % of wt mice serum)
Total IgE (mg/mL of serum)
d
No. of ILC2 cells
naive
Hp
% IL-13+ ILC2 cells
e
% GATA3+ of CD3+ CD4+ cells
% c-MAF+ of CD3+ CD4+ cells
GATA3
C-Maf
51%
49%
4%
14%
STAT6
Load
wt
cre
f
naive-wt
naive-cre
Th2-cre
Th2-wt
Fold change KO (log2 RPM)
Fold change WT (log2 RPM)
Log fold change
Mean expression
EGFR expression (AU)
Th2
g
Number of cells
Hours after activation
WT 0
WT 20ng/ml
WT 200ng/ml
Cre 0
Cre 20ng/ml
Cre 200ng/ml

Supplementary Figure S3. Related to Figure 1.

Characterization of the immune response of EGFR$^{\Delta CD4}$ mice to *H. polygyrus* infection. a-d) *wt* and EGFR$^{\Delta CD4}$ mice were infected with *H. polygyrus* larvae or left untreated. Samples were collected on day 14 after infection. **a)** FACS analysis of the expression of EGFR on CD4^{+} T-cells **(upper)** and macrophages **(lower)** from mLNs. **b)** Frequency of total CD4^{+}T helper **(upper)** and Th2 **(lower)** cells from mLNs of infected mice. **c)** Total IgE and *H. polygyrus*-specific, IgA and IgG1 titers in faeces and sera of infected mice. **d)** Frequency of ILC2 from mLNs of naïve and infected **(upper)** mice and the capability of ILC2 from mLNs of infected mice to induce IL-13 in response to IL-33 **(lower)**. **e-f)** Th2 cells from *wt* and EGFR$^{\Delta CD4}$ mice were generated in vitro by adding IL-2, IL-4 and neutralizing IFN-γ antibody into the culture**. e)** The expression of GATA3 and c-Maf was determined by FACS analysis and the expression of STAT6 was determined by WB. **f)** RNA-seq analysis of *wt* and EGFR-deficient in vitro differentiated Th2 cells before and after 4 day differentiation. Heatmap shows median centered, log2 RPKM gene expression values with yellow depicting maximal z-score of 3 log2 RPKM and blue depicting z-score of -3 log2 RPKM. MA plot shows (lack of) genotype specific gene expression changes after 4 day differentiation. **g)** The proliferation in response to various concentrations of IL-2 was compared between naïve T cells from *wt* and EGFR$^{\Delta CD4}$ mice. $^{**}P < 0.01$, compared as indicated (Two-tailed Mann–Whitney test). Data are representative of two independent experiments (mean ± SEM); results for individual mice are shown as dots.

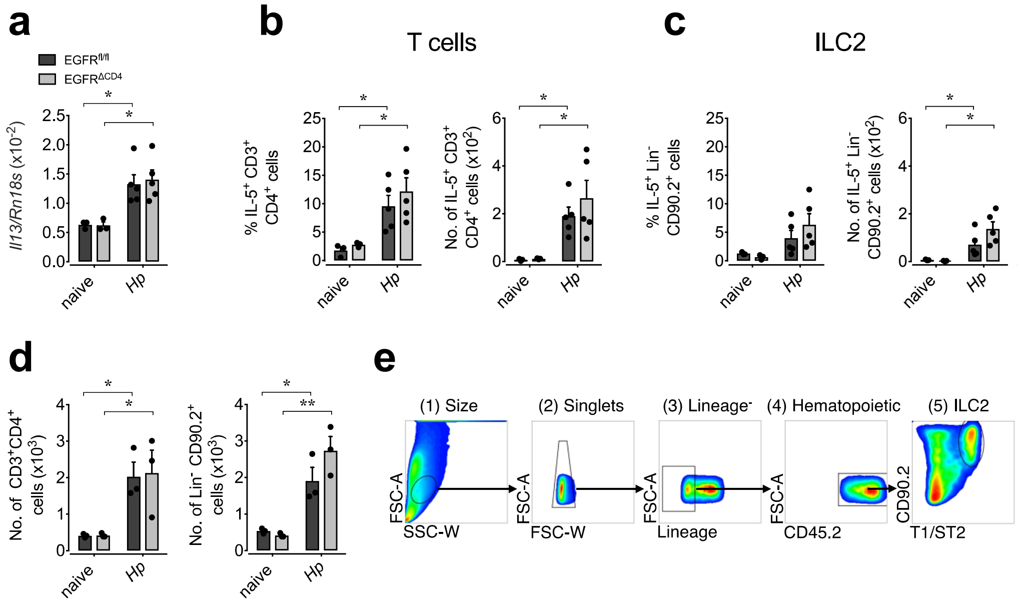

Supplementary Figure S4. Related to Figure 2.

Characterization of the cellular sources of IL-13 at the site of infection. *wt* and EGFR$^{\Delta CD4}$ mice were infected with *H. polygyrus* larvae or left untreated and samples were collected at day 8 post-infection. **a)** *Il13* was analyzed in the mLNs of naïve and infected mice by RT-PCR. **b-e)** Mice were treated with BFA 6 hours prior to harvest, after which, single cell preparations from duodena of naïve or infected mice were prepared and analyzed by FACS: Percentage and absolute number of IL-5 expressing T cells (**b**) and ILC-2 (**c**) as well as total T cells and ILC-2 **(d)**. **e)** Sequential identification of ILC-2 by flow cytometry. Cells are first identified by their size (1), and then as singlets (2). Lin− cells (3) are obtained by gating out CD3$^+$, CD11b$^+$, CD11c$^+$, FcεR1$^+$, CD19$^+$, and NK1.1$^+$ cells. Subsequently, hematopoietic cells are selected by their expression of CD45.2 (4). Finally, Lin− and CD45.2$^+$ populations are further subgated on the basis of the expression of CD90.2 versus T1/ST2. CD90.2$^+$ and T1/ST2$^+$ cells are what we defined as ILC-2 (5). All shown gates are children of the parent gates shown previously. In some experiments, CD3 was removed from the Lineage cocktail and used with a different conjugate in order to co-stain T-cells (CD3$^+$ CD4$^+$) and ILC-2. *$P < 0.05$, **$P < 0.01$ and ***$P < 0.001$ compared as indicated (ANOVA with the Tukey-Kramer HSD multiple-comparison test). Data are representative of two independent experiments (mean ± SEM); results for individual mice are shown as dots.

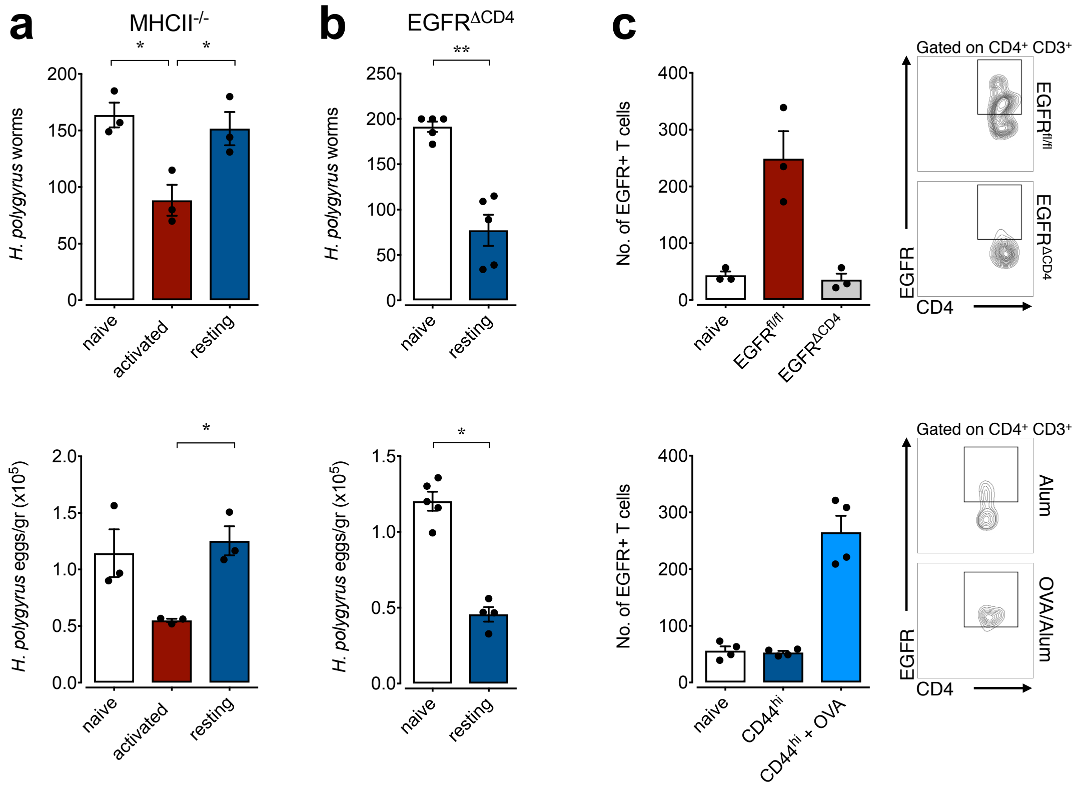

Supplementary Figure S5. Related to Figure 5.

Activated but not resting T-cells provide protection independently of TCR signaling. a) MHC-II$^{-/-}$ and **b)** EGFR$^{\Delta CD4}$ mice were infected with *H. polygyrus* larvae and 7 days post-infection received purified CD4^{+} T-cells derived from mLNs of naïve or *wt* mice that were infected with *H. polygyrus* for two weeks (activated in red) or from *H. polygyrus*-infected mice that had been treated with pyrantel embonate two weeks prior to transfer (resting in blue). Worm burden (**upper**) and egg counts in faeces (**lower**) were analysed 2 weeks post-transfer. **c)** EGFR$^{\Delta CD4}$ mice were infected with *H. polygyrus* larvae and received either FACS-sorted CD4^{+} CD69^{+} activated T-cells **(upper)** or FACS-sorted memory T-cells **(lower)** from mice that were challenged intra-nasally with OVA. EGFR expressing cells were recovered in single cells suspensions from the duodena of recipient mice and analyzed by FACS. *P < 0.05 and **P < 0.01, compared as indicated (Mann-Whitney U test). Data are representative of two independent experiments (except from c) (mean ± SEM); results for individual mice are shown as dots.

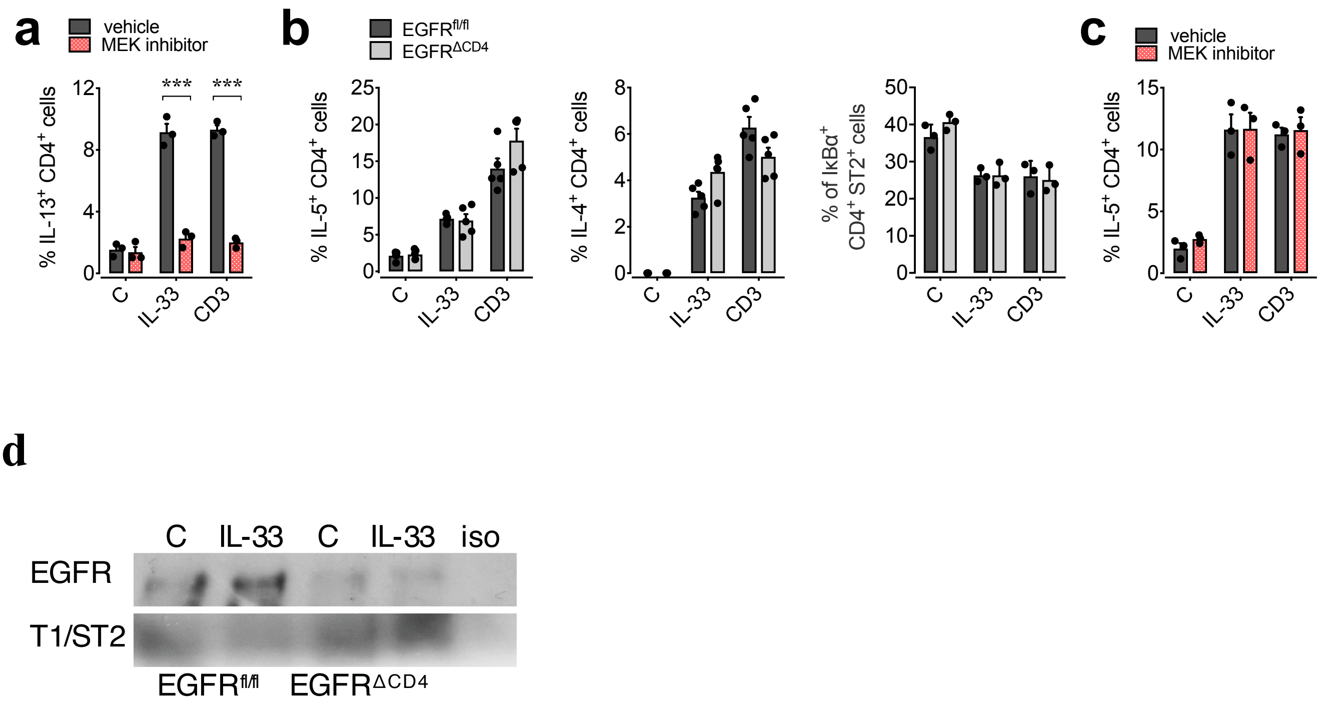

Supplementary Figure S6. Related to Figure 6.

Characterization of the signaling pathways required to induce IL-13 and IL-5 by Th2 cells. *wt* and EGFR$^{\Delta CD4}$ mice were infected with *H. polygyrus* larvae and on day 14 post-infection mLN were harvested. mLN cells were stimulated with IL-33, anti-CD3 or media in the presence **(a,c)** or absence **(b)** of MEK inhibitor (10 µM) (orange bars) and vehicle. **a)** Expression of IL-13 in *wt* T cells treated with MEK inhibitor was determined by FACS. **b**) Expression of IL-5, IL-4 and IκBα was determined by FACS analysis in *wt* and EGFR$^{\Delta CD4}$ T-cells. **c)** Expression of IL-5 in *wt* T cells treated with MEK inhibitor was determined by FACS. **d**) T1/ST2 was immune-precipitated from MACS-enriched *wt* and EGFR$^{\Delta CD4}$ CD4 T-cells and the presence of EGFR in the immuno-complexes was analyzed by Western blot.

***P < 0.001, compared as indicated (ANOVA with the Tukey-Kramer HSD multiple-comparison test). All data are representative of at least two independent experiments (mean ± SEM); results for individual mice are shown as dots.

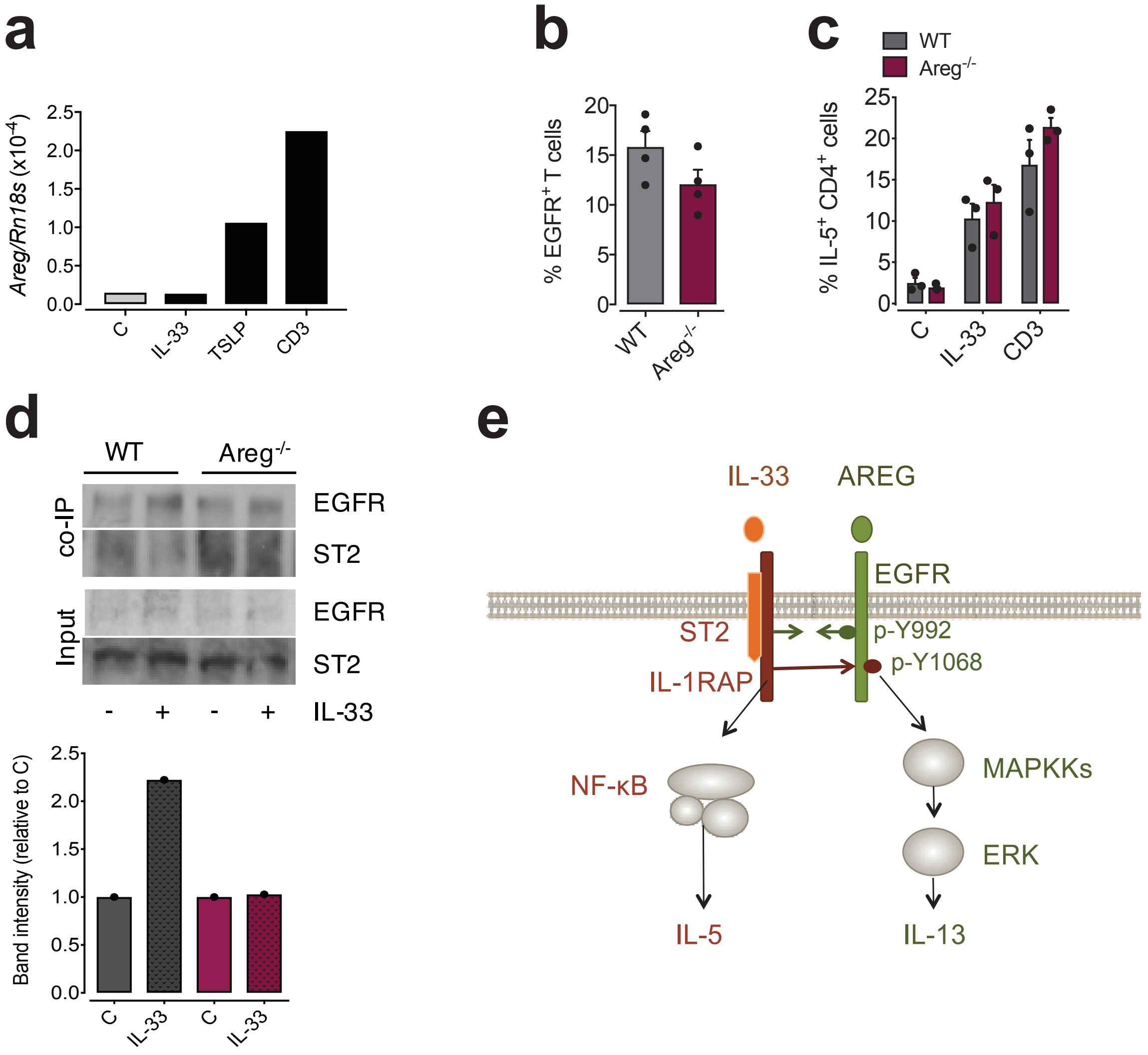

Supplementary Figure S7. Related to Figure 7.

Characterization of the function of AREG during the formation of an EGFR/ST2 signaling complex on the surface of Th2 cells. *wt* and *Areg*$^{-/-}$ mice were infected with *H. polygyrus* larvae and on day 14 post-infection mLN were harvested. **a)** AREG expression following different stimuli **b)** Comparison of EGFR expression on mLN CD4^{+} T cells from *H. polygyrus*-infected *wt* and *Areg*$^{-/-}$ mice. **c)** mLN cells were stimulated with IL-33, anti-CD3 or media only and expression of IL-5 was determined by FACS analysis. **d)** T1/ST2 was immune-precipitated from FACS sorted *wt* and *Areg*$^{-/-}$ CD4 T-cells and the presence of EGFR in the immuno-complexes was analyzed by Western blot (**upper**) and band intensity per lane determined (**lower**). **e)** Scheme explaining how AREG-induced Y-992 phosphorylation of the EGFR on Th2 cells allows for the formation of an active signaling complex between the EGFR and T1/ST2.

All data are representative of at least two independent experiments (mean ± SEM); results for individual mice are shown as dots.
